# Comorbidity of anxiety and dry eye disease: shared biomarkers and immune responses

**DOI:** 10.3389/fimmu.2026.1854853

**Published:** 2026-08-31

**Authors:** Xi Long, Xin Peng, Guicheng Liu, Pengfei Jiang, Pei Liu, Jun Peng, Qinghua Peng

**Affiliations:** 1 Hunan University of Chinese Medicine, Changsha, China; 2 The First Affiliated Hospital of Hunan University of Chinese Medicine, Changsha, China; 3 Quzhou Hospital of Zhejiang Medical and Health Group, Quzhou, China

**Keywords:** anxiety, circadian rhythm, comorbidity, dry eye disease, immune response

## Abstract

**Background:**

Dry eye disease (DED) and anxiety are prevalent disorders that often coexist, yet the molecular mechanisms underlying their comorbidity remain inadequately understood. This study sought to elucidate the convergent genes, pathways, and immune signatures that link DED and anxiety by employing integrated bioinformatics and experimental validation approaches.

**Methods:**

Datasets (GSE44101, GSE98793) were retrieved from the GEO database. Weighted gene co-expression network analysis (WGCNA), differential expression analysis, and LASSO regression were utilized to identify candidate genes associated with comorbidity. Subsequently, functional enrichment analysis, protein–protein interaction network analysis, immune infiltration deconvolution, and single-cell RNA sequencing were conducted. A comorbid DED-anxiety mouse model was developed using a combination of desiccating stress, 0.2% benzalkonium chloride, and chronic unpredictable mild stress. The expression of hub genes was validated through reverse transcription quantitative polymerase chain reaction(RT-qPCR) and Western blotting.

**Results:**

Researchers identified 128 candidate genes enriched in pathways like circadian rhythm. Four key genes (*BTBD1*, *BANK1*, *UBA3*, *NEDD1*) were highlighted for their expression in corneal epithelium cells. Immune analysis linked these genes to plasma cells and B cell subsets. In a mouse model, symptoms of dry eye, anxiety, inflammation, and neurotransmitter imbalance were observed, with the hub genes upregulated. RT-qPCR and Western blot analyses confirmed significant upregulation of *BTBD1*, *BANK1*, *UBA3*, and *NEDD1* in corneal tissues, while in the lacrimal gland and hippocampus, *BANK1* was elevated but *BTBD1*, *UBA3*, and *NEDD1* were downregulated, indicating tissue-specific regulatory patterns.

**Conclusion:**

The study suggests *BTBD1*, *BANK1*, *UBA3*, and *NEDD1* are linked to immune-inflammatory features in DED-anxiety comorbidity, with distinct tissue-specific expression profiles observed across the cornea, lacrimal gland, and hippocampus, offering a basis for future research into shared pathways.

## 1 Introduction

Dry eye disease (DED) is a complex, multifactorial condition affecting the tear film and ocular surface. It is characterized by symptoms of discomfort and visual disturbances, alongside tear film instability, hyperosmolarity, and inflammation of the ocular surface, which may ultimately result in tissue damage (1–3). Epidemiological research indicates that between 5% and 50% of the population is affected, with significant variation observed across different regions and age groups (4, 5). DED results from a combination of local ocular conditions, such as blepharitis and meibomian gland dysfunction, as well as systemic diseases including diabetes mellitus, Sjögren’s syndrome, rheumatoid arthritis, and systemic lupus erythematosus. This condition is associated with a substantial decline in vision-related quality of life and mental well-being 6, 7). According to the Diagnostic and Statistical Manual of Mental Disorders, Fifth Edition (DSM-5), anxiety is characterized as an excessive and persistent anticipation of future threats, accompanied by symptoms such as muscle tension, hypervigilance, and avoidant behavior (8). Anxiety disorders, which include specific phobia, social anxiety disorder, generalized anxiety disorder, panic disorder, agoraphobia, and childhood-onset conditions such as separation anxiety and selective mutism, represent the most prevalent category of psychiatric disorders globally. These disorders are marked by high chronicity and frequent comorbidity (9). They significantly contribute to global disability and economic costs, occurring 1.3–2.4 times more often in women than men (10, 11). Given their high prevalence and chronic burden, the clinical co-occurrence of DED and anxiety warrants further investigation.

In DED, environmental and endogenous factors contribute to tear film instability and hyperosmolarity, subsequently initiating both innate and adaptive immune responses at the ocular surface (12). This process involves antigen-presenting cells, T helper 1 (Th1) and T helper 17 (Th17) cells, as well as a wide range of cytokines and chemokines, including interleukin-1β (IL-1β), interleukin-6 (IL-6), tumor necrosis factor-alpha (TNF-α), and interleukin-17 (IL-17). These elements collectively is accompanied by a self-sustaining inflammatory cascade that results in damage to the corneal epithelium, lacrimal gland, and ocular nerves (13, 14) Anxiety disorders have also been associated with dysregulation of limbic–cortical circuits, activation of the hypothalamic–pituitary–adrenal axis, altered monoaminergic and GABAergic neurotransmission, and low-grade systemic inflammation (15). Patients with anxiety have been reported to exhibit elevated circulating levels of pro-inflammatory cytokines such as IL-1β, IL-6, and TNF-α, along with altered microglial activation, suggesting a bidirectional interaction between immune signaling and neural circuits involved in the regulation of fear and arousal (16). Importantly, many of the mediators and pathways implicated in DED also overlap with those involved in anxiety, indicating shared neuroendocrine-immune mechanisms. These observations suggest that systemic inflammation, stress-induced immune dysregulation, hypothalamic-pituitary-adrenal axis activation, sensory nerve sensitization, and neuroimmune communication may constitute shared biological substrates connecting anxiety with DED.

Several studies have found links between DED and psychiatric disorders like depression and anxiety (17), with DED symptoms often correlating with these mental health issues (18–20). Anxiety is particularly common among patients with eye conditions, highlighting the need for effective management. Research in this area could reflect new treatment strategies for both DED and anxiety (21). Animal studies have also investigated DED and anxiety-related phenotypes largely within separate experimental paradigms. Animal studies have largely investigated DED and anxiety-related phenotypes within separate experimental paradigms, with DED models focusing on ocular surface pathology and anxiety models on behavioral and neurobiological outcomes (22, 23). However, few studies have combined these paradigms to evaluate ocular surface pathology, anxiety-like behavior, and central neurobiological alterations within the same experimental framework. As a result, it remains unclear whether candidate molecular alterations associated with DED and anxiety are concordant or tissue-specific across the cornea, lacrimal gland, and hippocampus.

For DED, transcriptomic investigations have largely concentrated on ocular surface tissues, lacrimal gland alterations, or immune-related changes in local ocular inflammation (24, 25). For stress- and anxiety-related phenotypes, molecular studies have examined limbic and cortical regions involved in emotional regulation, including the hippocampus, amygdala, and prefrontal cortex (26, 27). Direct cross-tissue comparisons between the cornea and hippocampus remain scarce. As a result, it remains unclear whether DED and anxiety share candidate molecular signatures and whether these candidates exhibit coordinated or tissue-specific expression patterns across ocular and central tissues. Despite growing evidence linking DED with anxiety, several key questions remain unresolved. First, it remains unclear which candidate genes and biological pathways are potentially shared by DED and anxiety. Second, whether these candidates exhibit concordant or tissue-specific expression patterns across the cornea, lacrimal gland, and hippocampus has not been systematically examined. Third, the immune-cell signatures associated with the candidate molecular features shared by DED and anxiety remain incompletely characterized. In addition, *in vivo* studies simultaneously evaluating ocular surface injury, anxiety-like behavior, systemic inflammation, and central neurobiological alterations remain limited.

**Figure 1:**
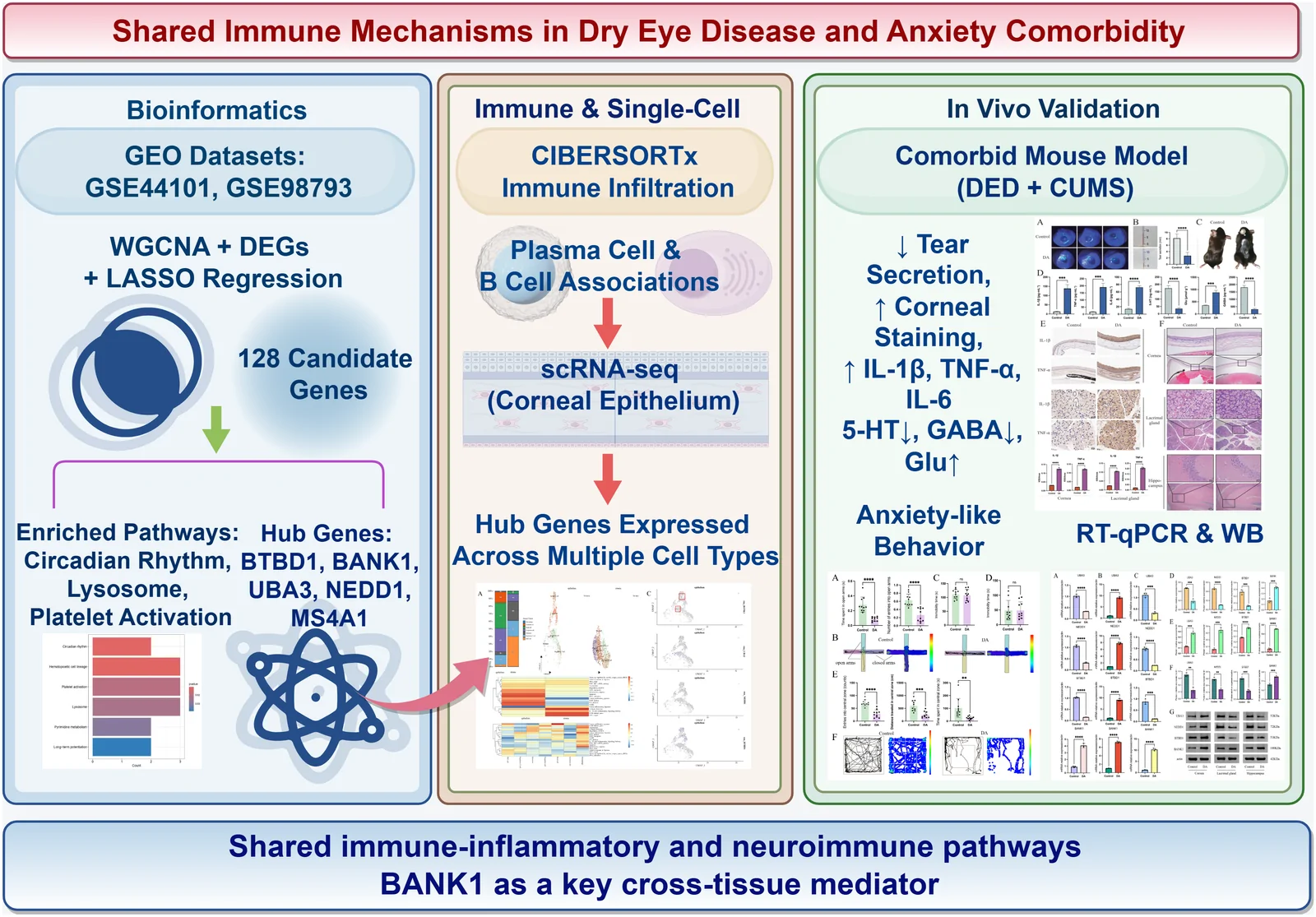
(Graphical abstract): Integrative bioinformatics and *in vivo* validation identified four hub genes (*BTBD1*, *BANK1*, *UBA3*, *NEDD1*) for DED-anxiety comorbidity, enriched in circadian rhythm pathways. The comorbid model confirmed DED and anxiety phenotypes and neurotransmitter imbalance, with corneal upregulation of hub genes and pan-tissue *BANK1* elevation. Shared immune-inflammatory pathways link the two conditions, highlighting *BANK1* as a key mediator. This figure was created using Figdraw (https://www.figdraw.com/static/index.html#/).

Circadian rhythm dysregulation represents a plausible but insufficiently examined biological process relevant to both DED and anxiety. Circadian regulation influences immune activity, neuroendocrine signaling, sleep-wake behavior, tear secretion, and ocular surface homeostasis (28). Accordingly, circadian dysregulation could be associated with both ocular surface abnormalities and anxiety-related neuroimmune changes. Assessing the relationship of circadian rhythm dysregulation and immune-cell infiltration patterns may provide an integrated view of biological processes potentially shared by the two conditions. Given these unresolved questions, the present study aimed to bridge the knowledge gap by using integrated bioinformatics and *in vivo* validation in a DED-anxiety mouse model, focusing on immune-related genes and the potential relationship of circadian rhythm disruption and comorbidity of DED-anxiety, as shown in **Figure 1**.

## 2 Materials and methods

### 2.1 Bioinformatics

#### 2.1.1 Data source and preprocessing

The Gene Expression Omnibus (GEO) (http://www.ncbi.nlm.nih.gov/geo/) is a comprehensive and publicly accessible repository of gene expression data, developed and maintained by the National Center for Biotechnology Information (NCBI). This database houses high-throughput gene expression data submitted by research institutions globally. In our study, we utilized the search terms “dry eye” and “anxiety” to extract gene expression datasets pertinent to DED and anxiety. The inclusion criteria for dataset selection were as follows: (1) each group must have a sample size of at least three, (2) samples from both normal and control groups must be included, and (3) the dataset must provide access to raw and complete data that can be exported for analysis. Following the dataset screening, we preprocessed the matrix files obtained from the platform, aligning the probes with their corresponding gene symbols based on the platform’s annotation files. Ultimately, we generated gene matrices with gene symbols as row identifiers and sample names as column identifiers for subsequent analyses. All bioinformatics analyses were performed using R software (version 4.2.0) with the following packages: limma (3.6.9), WGCNA (1.74), glmnet (5.0), clusterProfiler (4.0.2), org.Hs.eg.db (3.20.0), Seurat (5.5.1), ggplot2 (3.4.0), pheatmap (1.0.13), and corrplot (0.95).

#### 2.1.2 Weighted correlation network analysis

WGCNA employs a gene network construction algorithm that considers biological functions holistically by evaluating the expression similarity between genes. In our study, to identify gene co-expression modules associated with DED and anxiety, we utilized gene expression profiles to exclude the top 50% of genes exhibiting the smallest Median Absolute Deviation (MAD), a robust variance measure recommended for filtering non-informative genes (29). Outlier genes and samples were removed using the “good Samples Genes” function from the WGCNA R package. This package facilitated the construction of scale-free co-expression networks through a power function, which was used to develop a weighted adjacency matrix. The parameter β serves as a soft-threshold, accentuating strong inter-gene correlations while diminishing weaker ones. Genes with analogous expression profiles were clustered into gene modules, with the minimum module size set at 30 and sensitivity at 3. For further module analysis, we calculated the similarity of genes within modules, determined the cut lines for module dendrograms, and merged modules with distances less than 0.25. Modules exhibiting high correlation coefficients with DED and anxiety were selected, and genes from these modules were extracted for subsequent analysis.

#### 2.1.3 Identification of differently expression genes

DEGs associated with DED and anxiety were identified utilizing the limma package within the R software environment. Initially, datasets were processed and transformed into expression matrices, followed by appropriate grouping. Subsequent normalization and analysis were conducted using the limma package, with significance thresholds set at an adjusted *P*-value of less than 0.05 and a log2 fold change (|FC|) of at least 1.2. This analysis facilitated the identification of DEGs, providing insights into their upregulation and downregulation patterns. By employing a Venn diagram to examine the intersection of upregulated and downregulated genes, we identified genes that are common to both DED and anxiety.

#### 2.1.4 Machine learning analysis

To further explore the diagnostic value of the intersecting genes, we constructed a predictive model of reliability using machine learning. Regression analysis was performed using the least absolute shrinkage and selection operator (LASSO) with the glmnet package in R. Gene expression values were used as predictor variables and the sample groups as response variables. Ten-fold cross-validation (cv) was performed using the cv.glmnet function, and the optimal λ value was selected as λ min based on the minimum mean cross-validated error. The cross-validation error curves were plotted to determine the optimal regularization parameters. Based on the coefficients of the Lasso regression, genes with non-zero coefficients were identified as the selected signature genes.

#### 2.1.5 Enrichment analysis and functional annotation

A Venn diagram, facilitated by the online tool available at https://bioinfogp.cnb.csic.es/tools/venny/, was utilized to identify the intersection of the genes obtained. This approach enabled the identification of genes co-expressed in DED and anxiety through WGCNA, DEGs, and machine learning analysis. To further elucidate the functions of the identified genes, KEGG enrichment analysis and Gene Ontology (GO) functional annotation were conducted on genes associated with the comorbidity of DED and anxiety. For gene set functional enrichment analysis, the KEGG REST API (https://www.kegg.jp/kegg/rest/keggapi.html) was employed to access the latest gene annotations from the KEGG Pathway. The R package clusterProfiler (version 3.14.3) was used to perform the enrichment analysis. The analysis incorporated GO annotations from the R package org.Hs.eg.db (version 3.1.0), encompassing biological processes (BP), cellular components (CC), and molecular functions (MF), to derive gene set enrichment results. The analysis parameters were set with a minimum gene set size of 5 and a maximum of 5000, with statistical significance determined at *P* < 0.05 and a Benjamini-Hochberg (BH) false discovery rate (FDR) < 0.1.

#### 2.1.6 Protein-protein interaction network construction and modular analysis

To elucidate the interactions among genes identified through WGCNA and DEG analyses, the STRING database (https://string-db.org) was utilized to construct a PPI network. The analysis was conducted with the species parameter set to “Homo sapiens.” The resulting protein data were subsequently imported into Cytoscape (http://www.cytoscape.org, version 3.8.2) for comprehensive PPI network analysis and visualization. Network analysis was executed using the “Analyze Network” tools within Cytoscape. Key functional modules within the PPI network were identified using the MCODE plug-in, which facilitated the extraction of closely interacting subnetworks. The specific parameters employed for this analysis included a degree cutoff of 2, a maximum depth of 100, a node score cutoff of 0.2, and a K-core of 2.

#### 2.1.7 Identification and analysis of hub genes

The PPI genes were examined utilizing the degree-value algorithm through the CytoHubba plugin to ascertain the significance of individual genes within the network and to identify hub genes. The analysis employed MCC, DMNC, and Degree values as reference standards, in conjunction with the results from MCODE, to determine hub genes. Subsequently, GeneMANIA (https://genemania.org/) was employed to generate co-expression networks for the identified hub genes.

#### 2.1.8 Validation and Immune infiltrate analysis

We conducted validation using an independent dataset analyzed with R, considering *P*-values less than 0.05 as statistically significant. CIBERSORTx, a computational tool developed by the Alizadeh and Newman Labs, was employed to impute gene expression profiles and estimate the abundances of 22 distinct immune cell types within a heterogeneous cell population (30). The CIBERSORT method was selected for its efficacy, and analyses were performed utilizing the R software packages ggstatsplot and ggplot2. This approach enabled us to evaluate the exploratory correlations between shared characteristic genes and immune cell infiltration within the tissue microenvironment using Spearman correlation tests.

#### 2.1.9 The single-cell RNA sequencing analysis

For the single-cell RNA sequencing analysis, data were sourced from the GEO database. The Seurat package was utilized to construct the object and filter out low-quality cells. Standard data preprocessing protocols were implemented to evaluate the number of genes, number of cells, and mitochondrial content. Cells exhibiting fewer than 200 or more than 4,000 detected genes, as well as those with elevated mitochondrial content (>20%), were excluded from further analysis. Following log-normalization, normalization was applied to each cell. A set of highly variable genes (HVGs), also referred to as “magnet genes,” which display significant differential expression between groups, were identified across all samples and employed for grouping and cell identification. Marker genes for each cluster were determined based on their unique expression patterns within the respective cell types. To define a cluster as a distinct population, a minimum of two additional characteristic genes were required. Once all cell populations were identified based on annotation results, the cell populations were renamed accordingly. Subsequently, variations in metabolic features across different tissues were examined, alongside the distribution of cellular expression.

### 2.2 *In vivo* experiment

#### 2.2.1 Chemicals and reagents

Benzalkonium chloride (Shanghai Mclain Biochemical Technology Co., Ltd., Licence No. C14501094), Sodium pentobarbital (Sigma-Aldrich, USA, batch no. 2410116),phenol red cotton thread (Tianjin Jingming New Technology Development Co., Ltd., Registration No. 20250601). Sodium fluorescein (GLPBIO Technology LLC, USA, batch no. 100511-0725),Mouse IL-1β enzyme-linked immunosorbent assay (ELISA) Kit (Hangzhou Hua’an Bio, batch number EM00290001), Mouse TNF-α ELISA Kit (Hangzhou Hua’an Bio, batch number EM00100024), IL-6 ELISA kit (Huaan Biological, Cat. No. EM0004), 5-hydroxytryptamine (5-HT) ELISA kit (Wuhan Feien Biotechnology Co., Ltd., Cat. No. EM1465), gamma-aminobutyric acid (GABA) ELISA kit (Wuhan Feien Biotechnology Co., Ltd., Cat. No. EM1603), glutamate (Glu) ELISA kit (Nanjing Jiancheng Bioengineering Institute Co., Ltd., Cat. No. A074-1-1).BTBD1 Polyclonal antibody (AWA55139), BANK1 Rabbit pAb (AWA67252), UBA3 Mouse Monoclonal Antibody (AWA04334), NEDD1 Polyclonal antibody (AWA70631).

#### 2.2.2 Experimental animals

Twenty healthy, clean-grade female C57BL/6J mice, weighing between 18 and 20 grams and aged 6 to 8 weeks, were procured from Hunan Sileike Jingda Experimental Animal Co., Ltd. (Hunan, China). The mice were housed at the Laboratory Animal Center of Hunan University of Chinese Medicine under controlled environmental conditions, including a room temperature of 25 ± 3 °C, relative humidity of 50%–70%, and a 12-hour light/dark cycle (lights on from 06:00 to 18:00). They had unrestricted access to standard chow and water in a quiet setting. Upon arrival, the mice underwent a one-week acclimatization period prior to the commencement of experimental procedures. The study protocol received approval from the Animal Ethics Committee of Hunan University of Chinese Medicine, adhering to the ethical standards for animal experimentation at the institution (ethics number: HMCCM21-2501-27). All experimental procedures were conducted in compliance with the “Guide for the Care and Use of Laboratory Animals” and the ARRIVE 2.0 guidelines. At the conclusion of the experiment, the mice were anesthetized and euthanized via intraperitoneal injection of 1% sodium pentobarbital solution (0.4 mL per mouse), following the American Veterinary Medical Association (AVMA) Guidelines for the Euthanasia of Animals.

#### 2.2.3 Experimental design

Twenty C57BL/6J mice were procured and permitted a seven-day acclimatization period. Subsequently, the animals were randomly allocated to either a normal control group or a model group, with each group comprising ten mice, through the use of a random number table. The mice assigned to the model group underwent a benzalkonium chloride (BAC)-based DED induction protocol in conjunction with chronic unpredictable mild stress (CUMS) to develop a comorbidity model of DED and anxiety. In contrast, the mice in the normal control group did not receive any treatment.

In the model group, DED was experimentally induced by exposing the mice to a controlled dry chamber environment, characterized by a relative humidity of less than 30% and an airflow velocity of 2.2 ± 0.2 m/s. Additionally, a 0.2% BAC solution, at a volume of 5 μL per eye, was administered twice daily over a period of 28 consecutive days. Anxiety-like behavior was elicited through a CUMS protocol, adapted with minor modifications from the methodology outlined by Yanqin Luo. The protocol incorporated a series of stressors: (1) 24-hour food deprivation; (2) 24-hour water deprivation achieved by removing the drinking bottles; (3) 12 hours of continuous overnight illumination; (4) a 20-minute tail pinch; (5) a 5-minute forced swim in cold water at temperatures ranging from 4 to 8 °C; (6) exposure to a novel environment for 24 hours; (7) placement of empty drinking bottles; and (8) noise interference. These stressors were administered once daily in a randomized sequence over four consecutive weeks, ensuring that no single stressor was repeated on consecutive days to prevent the mice from anticipating the type of stressor.

Upon completion of the modeling period, the severity of DED and anxiety-like behaviors were assessed. Tear secretion and corneal fluorescein staining were measured to evaluate ocular surface damage. Anxiety-like phenotypes were quantified using the elevated plus maze and open field test, while depressive-like behaviors were detected using the forced swim test and tail suspension test. the successful establishment of the dry eye disease and anxiety comorbidity model (DA) was confirmed when these physiological and behavioral indices exhibited significant differences compared to the normal control group. Mice in the control group were maintained under standard conditions with a relative humidity of 50–70%.

Upon completion of the experiment, mice from each group were anesthetized via intraperitoneal injection of 1% pentobarbital sodium at a dosage of 0.4 mL per mouse. Serum concentrations of the pro-inflammatory cytokines IL-1β, TNF-α, and IL-6, along with hippocampal levels of 5-HT, GABA, and Glu, were measured utilizing enzyme-linked immunosorbent assay ELISA in accordance with the manufacturer’s protocols. Samples of the cornea and lacrimal gland were collected. Tissues from three randomly selected mice in each group were fixed in 4% paraformaldehyde for hematoxylin and eosin (H&E) staining. Corneal and lacrimal gland samples from an additional three mice per group were prepared for immunohistochemistry (IHC). The remaining corneal and lacrimal gland tissues were rapidly frozen and stored at –80 °C for subsequent analyses via Western blot (WB) and real-time quantitative reverse transcription polymerase chain reaction (RT-qPCR).

#### 2.2.4 Tear secretion measurement and corneal fluorescein staining

On the day subsequent to the final intervention, tear secretion was evaluated. The lower eyelid was carefully retracted, and a phenol red thread was manipulated using forceps. Approximately 1 mm of the thread was positioned on the conjunctival surface at one-third of the distance from the lateral canthus and was maintained in place for 15 seconds. The length of the moistened and red-stained segment of the thread was subsequently measured under a microscope and recorded in millimeters. A 1% fluorescein sodium solution (1 μL) was instilled into the conjunctival sac, and excess dye was removed with absorbent paper. After a 10-second interval, the mice were manually prompted to blink three times. Corneal fluorescein staining was then examined using a slit-lamp microscope equipped with a cobalt blue filter.

#### 2.2.5 ELISA for IL-1β,TNF-α,IL-6, 5-HT, Glu, GABA levels

The levels of IL-1β, TNF-α, and IL-6 in serum, 5-HT in plasma, and Glu and GABA in hippocampal tissue homogenates were determined using commercially available ELISA kits in accordance with the manufacturers’ protocols. Briefly, standards and appropriately prepared samples were added to pre-coated 96-well plates and incubated with the corresponding detection antibodies and enzyme conjugates. After washing, TMB substrate was added for color development, and the reaction was terminated with stop solution. Absorbance was measured at 450 nm using a microplate reader, and the concentrations were calculated from standard curves and expressed as ng/mL, pg/ml and μmol/gprot.

#### 2.2.6 IHC detection of IL-1β and TNF-α in the cornea and lacrimal gland

IHC analysis was conducted to evaluate the expression of IL-1β and TNF-α in the corneal and lacrimal gland tissues. Tissues from one side of each mouse were embedded in paraffin, sectioned, deparaffinized, rehydrated, and subjected to heat-induced antigen retrieval using a sodium citrate buffer. Endogenous peroxidase activity was inhibited using 1% periodic acid. The sections were incubated overnight at 4 °C with primary antibodies specific to IL-1β and TNF-α, followed by incubation with an HRP-conjugated anti-rabbit IgG polymer secondary antibody. The signal was developed using DAB, counterstained with hematoxylin, dehydrated, and mounted. Positive staining, indicated by brown to dark-brown coloration, was assessed via light microscopy.

#### 2.2.7 H&E

H&E staining was employed to evaluate morphological alterations in the cornea and lacrimal gland. Tissues from one side of each mouse were fixed in 4% paraformaldehyde, subjected to a graded dehydration process, cleared, embedded in paraffin, sectioned, and subsequently stained with hematoxylin and eosin. Following dehydration and mounting with neutral resin, the sections were examined using light microscopy, and representative images were captured for the histopathological assessment of each tissue.

#### 2.2.8 Behavioral assessments

A comprehensive series of behavioral assessments was conducted to evaluate anxiety-like and stress-related behaviors in mice. These assessments included the tail suspension test, forced swim test, elevated plus maze, and open field test. All experiments were carried out in a quiet, dimly lit environment, and the apparatus was meticulously cleaned with 70% ethanol between trials to eliminate any olfactory cues. Prior to each assessment, the mice were given a 30-minute acclimation period to the testing environment.

- Tail Suspension Test (TST): In the tail suspension test, mice were affixed by the distal 1–2 cm of their tails using adhesive tape attached to a horizontal bar, allowing them to hang freely at a height of approximately 40–50 cm above the floor. Each trial had a duration of 6 minutes, during which the immobility time—characterized by the lack of active escape-oriented movements—was recorded. An increase in immobility time was interpreted as an indication of heightened stress or anxiety-like behavior.
- Forced Swim Test (FST): The forced swim test was administered utilizing a transparent cylindrical container with dimensions of 20–25 cm in height and 10–15 cm in diameter. The container was filled with water maintained at a temperature of 23–25 °C to a depth sufficient to prevent the mice from contacting the bottom. Each mouse was individually placed into the cylinder for a duration of 6 minutes. The immobility of the mice was assessed during the final 4 minutes, with immobility operationally defined as the minimal movements necessary to maintain the head above the water surface. Extended periods of immobility were interpreted as indicative of heightened behavioral despair or an increased stress burden.
- Elevated Plus Maze (EPM): The elevated plus maze was composed of two open arms and two closed arms, positioned approximately 50 cm above the ground. Each mouse was placed at the central junction of the maze, oriented toward an open arm, and permitted to explore freely for a duration of 5 minutes. The duration of time spent in the open arms and the frequency of entries into these arms were meticulously recorded. A decrease in activity within the open arms was interpreted as indicative of increased anxiety-like behavior.
- Open Field Test (OFT): The open field apparatus consisted of a square arena measuring 50 × 50 cm. Each mouse was gently placed at the center of the arena and permitted to explore for a duration of five minutes. Quantitative measurements included the total distance traveled and the time spent in the central zone. A decrease in central exploration and reduced locomotor activity were interpreted as indicative of heightened anxiety levels.

#### 2.2.9 RT-qPCR

Total RNA was isolated from mouse corneal and lacrimal gland tissues utilizing the TRIzol reagent, followed by cDNA synthesis employing a reverse transcription kit in accordance with the manufacturer’s protocol. Quantitative PCR was conducted on a real-time PCR system under the following thermal cycling conditions: an initial denaturation at 95 °C for 10 minutes, succeeded by 40 cycles of denaturation at 95 °C for 15 seconds, annealing at 60 °C for 30 seconds, and extension at 72 °C for 60 seconds. To verify the specificity of amplification, a melting curve analysis was performed over a temperature range of 60–95 °C. Relative mRNA expression levels were determined using the 2^−ΔΔCt method, with normalization to an internal reference gene, and expressed as fold changes relative to the control group, the primer design as shown in **Table 1**.

**Table 1:** Primer design.

Gene name	Forward primers (5′–3′)	Reverse primer (5′–3′)
NEDD1	TCAACCCTCACACGTCACC	AGTGGAACGGGCTTACATTTG
UBA3	CCTTCACACACCCCGATTTC	GCCTTGGGTCTTCCGACATC
BANK1	AGACTCAAGAGCCAAGTATCCT	TCACCAGGTTTCTCACATGGAA
BTBD1	ACCTTGGGACAGAATGATACTGG	TCAGTCCTTTTGTGCCATAGTG
actin	ACATCCGTAAAGACCTCTATGCC	TACTCCTGCTTGCTGATCCAC

#### 2.2.10 WB

Frozen corneal and lacrimal gland tissues underwent homogenization for the extraction of total protein, with protein concentrations quantified via the BCA assay. Equivalent protein quantities were resolved using SDS-PAGE and subsequently transferred onto nitrocellulose membranes through a wet transfer system. The membranes were blocked with 5% non-fat milk in PBST and incubated overnight at 4 °C with primary antibodies targeting UBA3, NEDD1, BTBD1, and BANK1 (1:500), and β-actin (1:5000). Following PBST washes, HRP-conjugated secondary antibodies were applied, and the bands were visualized using enhanced chemiluminescence. Densitometric analysis was conducted using Quantity One software, with target protein levels normalized to β-actin.

#### 2.2.11 Statistical analysis

Statistical comparisons between the two groups were performed using an unpaired two-tailed Student’s t-test. Quantitative data are presented as the mean ± standard deviation (SD), with statistical significance established at a *P*-value of less than 0.05.

## 3 Results

### 3.1 Bioinformatic screening

#### 3.1.1 GEO information

DED and anxiety were systematically retrieved from the GEO database. The datasets GSE44101 and GSE98793 were selected for primary analysis, while GSE40611 and GSE61672 were chosen for validation purposes. Additionally, GSE186433 was identified for single-cell RNA sequencing analysis. Detailed information regarding the datasets and demographic characteristics can be found in **Table 2**.

**Table 2:** Specific information on the source of the data.

Data type	GEO accession	Title	Platform	Organism	Group
Analysis	GSE44101	Expression data from Spdef +/+ and Spdef -/- mice	GPL126	Mus musculus	3 normal samples and 3 disease samples
Analysis	GSE98793	Gene expression analysis in whole blood samples obtained from donors diagnosed with MDD compared to healthy controls	GPL570	Homo sapiens	64 normal samples and 64 disease samples
Validation	GSE40611	Gene expression data of parotid tissue from Primary Sjogren's Syndrome and controls	GPL570	Homo sapiens	18 normal samples and 31 disease samples
Validation	GSE61672	Blood gene expression profiles associated with symptoms of generalized anxiety disorder	GPL10558	Homo sapiens	179 normal samples and 157 disease samples
Single-cell RNA sequencing	GSE186433	Single cell transcriptomics reveal the heterogeneity of the human cornea to identify novel markers of the limbus and stroma	GPL24676	Homo sapiens	12 human cornea samples

#### 3.1.2 WGCNA

**Figure 2:**
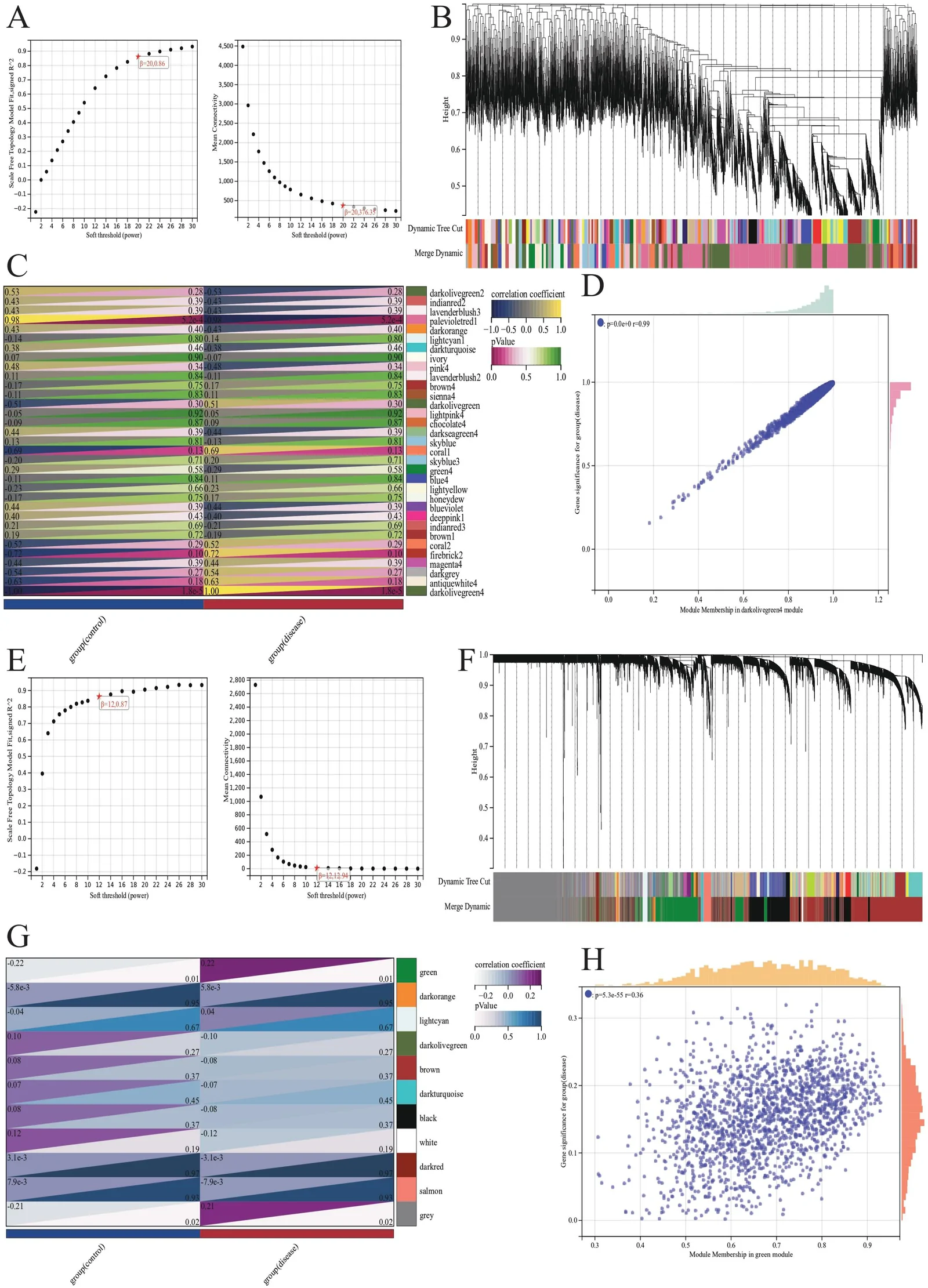
Weighted Gene Co-expression Network Analysis in DED and anxiety **(A)** Selection of soft thresholds (β) for WGCNA analysis in GSE44101 dataset. **(B)** Module division of the GSE44101 dataset. **(C)** Correlation within modules in the GSE44101 dataset. **(D)** Module most correlated with clinical traits in the GSE44101 dataset. **(E)** Selection of soft thresholds (β) for WGCNA analysis in GSE98793 dataset. **(F)** Module division of the GSE98793 dataset. **(G)** Correlation within modules in the GSE98793 dataset. **(H)** Module most correlated with clinical traits in the GSE98793 dataset.

Following preprocessing, data from the GSE44101 and GSE98793 datasets were analyzed to derive sample clusters. For the construction of a co-expression network, β values of 20 and 12 were selected as the soft threshold for the GSE44101 and GSE98793 datasets, respectively. These thresholds were chosen based on the following criteria: (1) a scale-free fitting index exceeding 0.8, (2) satisfactory average network connectivity, and (3) adherence to a non-scale topological network distribution. Module identification was conducted using a dynamic tree-cutting algorithm, followed by the merging of similar modules, resulting in gene sets within the modules that exhibited strong interrelations. Consequently, the GSE44101 dataset yielded 33 modules, while the GSE98793 dataset produced 11 modules. Upon correlating the modules from the GSE44101 and GSE98793 datasets with the clinical characteristics (treatment and control) of the samples, and applying thresholds of 0.8 for module membership (MM), 0.1 for gene significance (GS), and 0.1 for weight, it was observed that the darkolivegreen4 module of the GSE44101 dataset exhibited a high correlation with the clinical characteristics of the tissues (*r* = 0.99, *P* < 0.001). Similarly, the green module of the GSE98793 dataset demonstrated a strong correlation with clinical characteristics, as shown in **Figure 2**.

#### 3.1.3 DEGs and machine learning analysis

**Figure 3:**
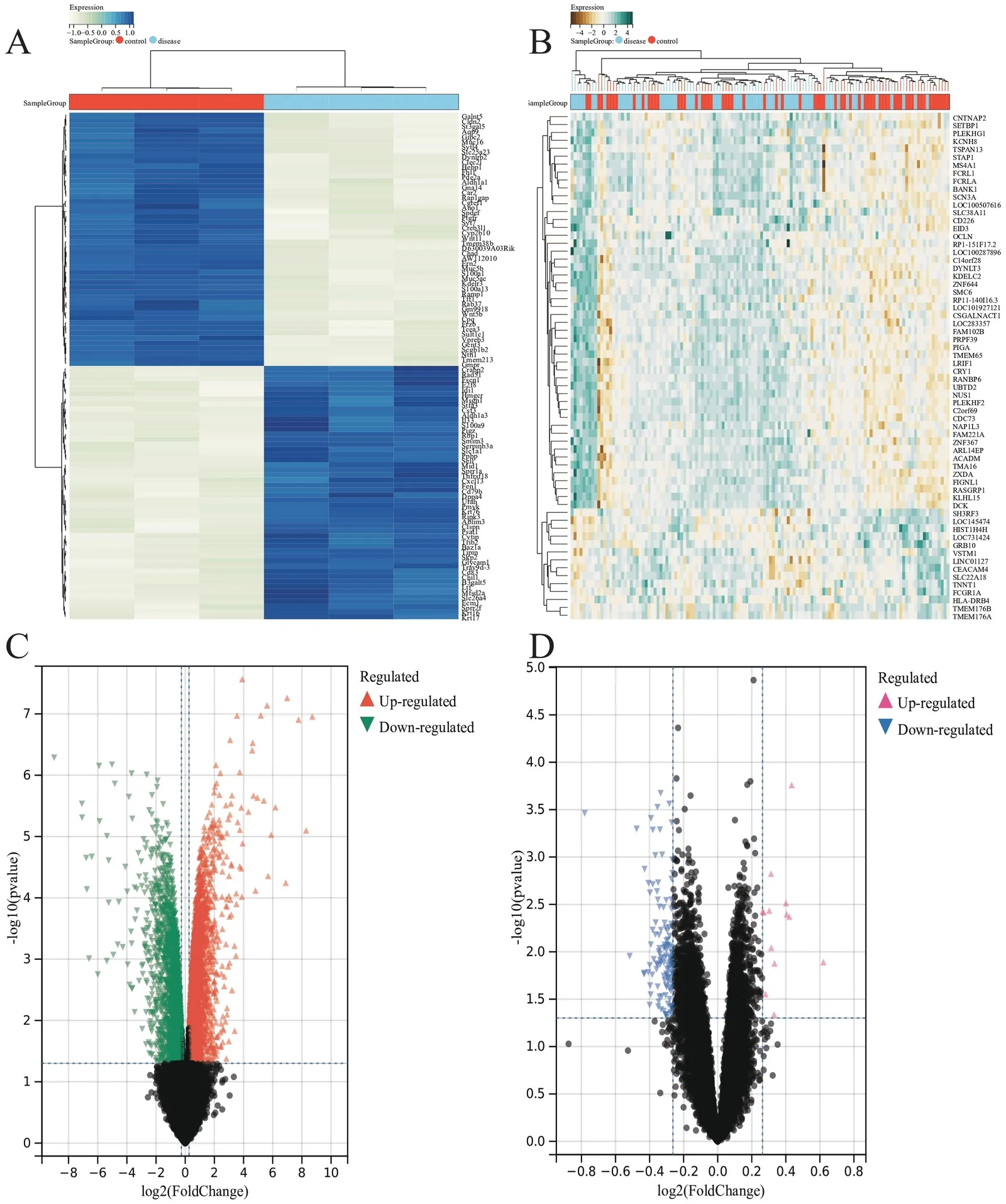
DEGs in DED and anxiety. **(A)** Heatmap of DEGs in the GSE44101 dataset. **(B)** Heatmap of DEGs in the GSE98793 dataset. **(C)** Volcano plot of DEGs in the GSE44101 dataset. **(D)** Volcano plot of DEGs in the GSE98793 dataset.

**Figure 4:**
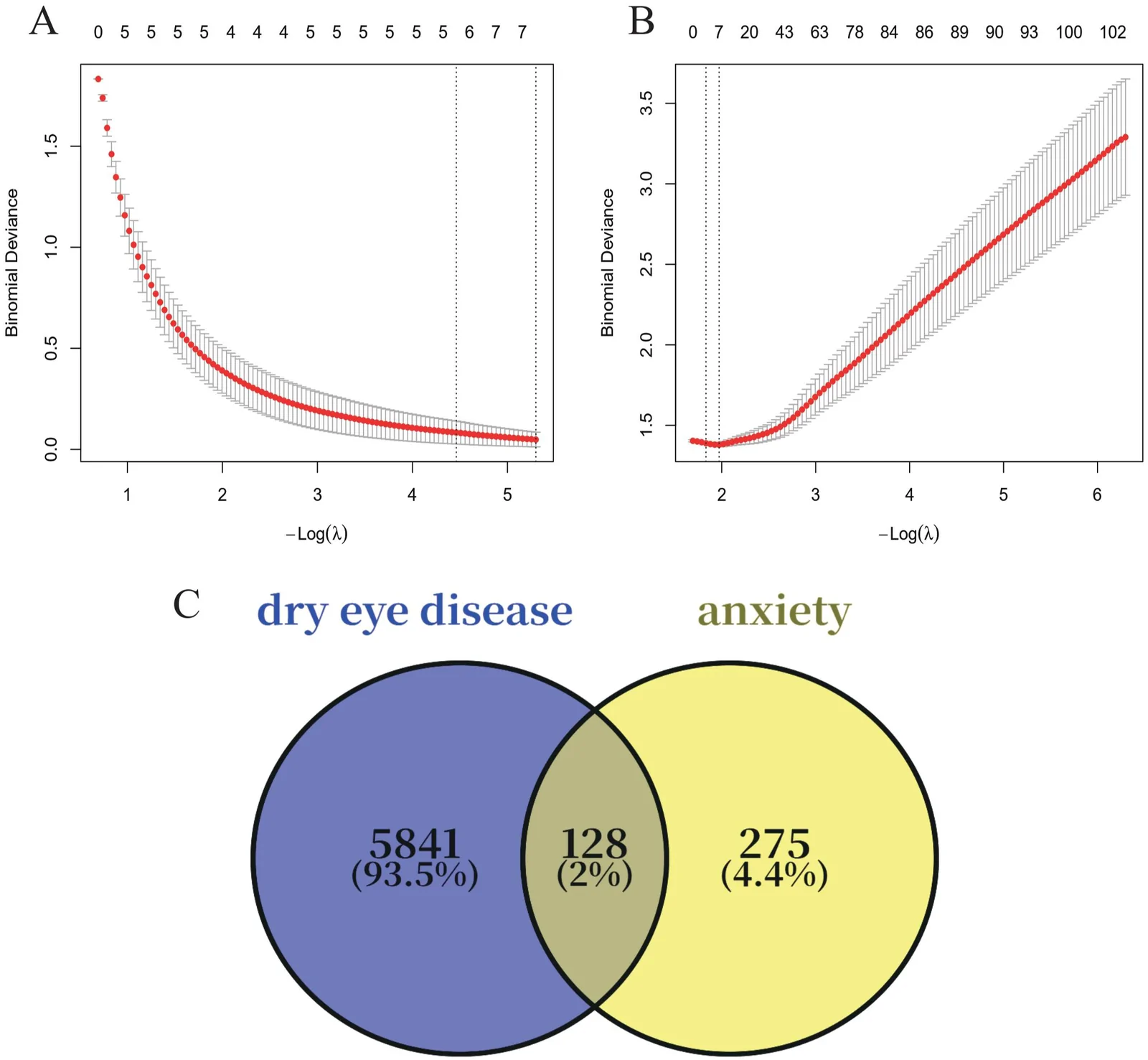
Machine learning analysis. **(A)** LASSO analysis in the GSE44101 dataset. **(B)** LASSO analysis in the GSE98793 dataset. **(C)** Venn diagram showing the intersection of two disease-associated gene lists. The blue circle represents the combined gene set for DED (GSE44101) derived from WGCNA, DEGs, and LASSO screening within that dataset. The yellow circle represents the combined gene set for anxiety (GSE98793) derived from WGCNA, DEGs, and LASSO screening within that dataset. The overlapping region (128 genes) represents the candidate genes shared between DED and anxiety.

Differential gene expression analysis was conducted on datasets GSE44101 and GSE98793, revealing 5,939 DEGs in GSE44101 and 135 DEGs in GSE98793, as illustrated in **Figure 3**. Subsequently, the LASSO regression algorithm was employed for further analysis. This analysis identified seven key genes in each dataset, GSE44101 and GSE98793, which were deemed characteristic genes with significant correlations to the samples, as depicted in **Figures 4A, B**.

#### 3.1.4 Enrichment analysis and functional annotation

**Figure 5:**
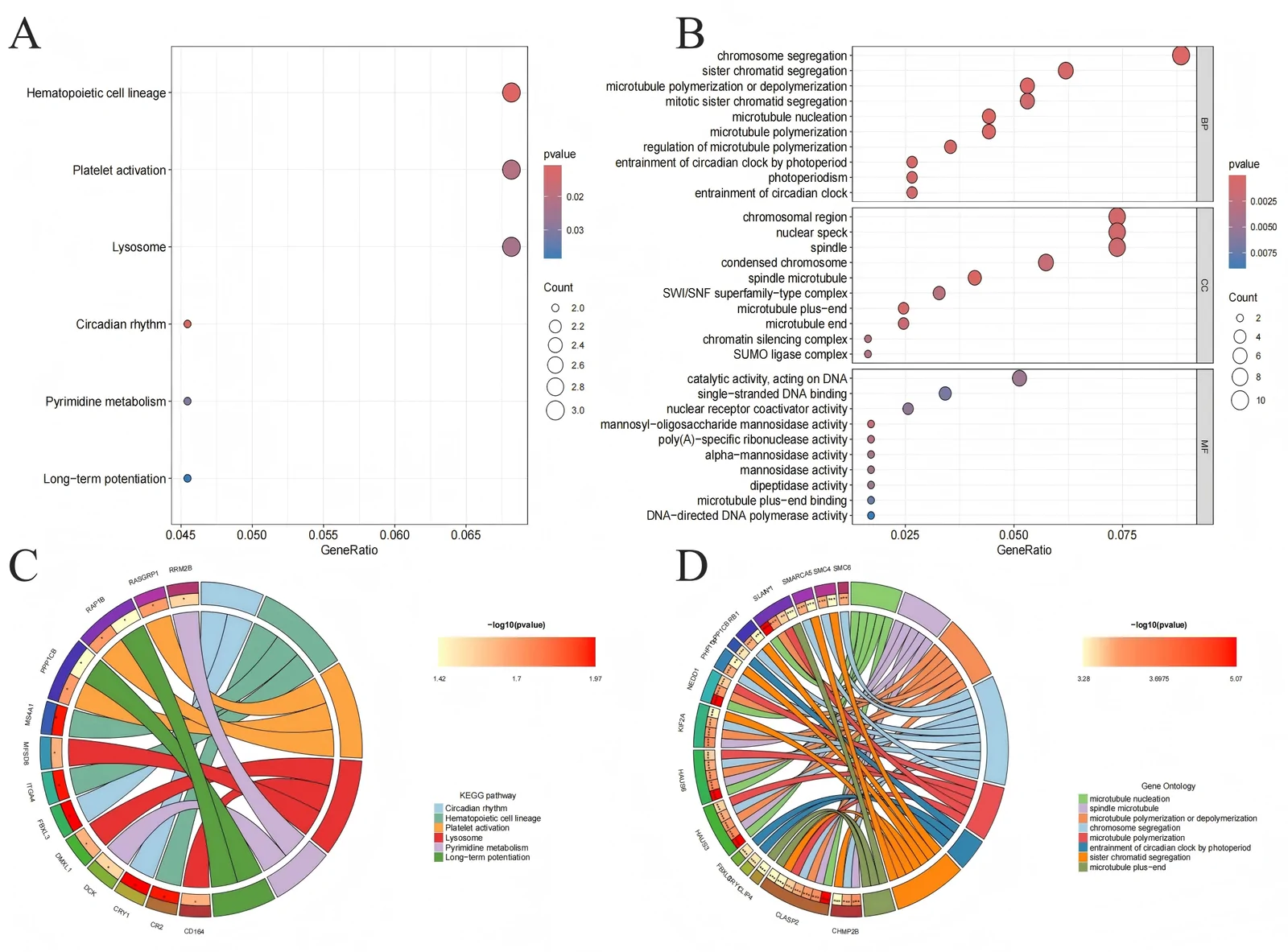
Enrichment analysis and functional annotation. **(A)** Bubble diagram of KEGG functional analysis. **(B)** Bubble diagram of KEGG enrichment analysis. **(C)** Circle diagram of KEGG functional analysis. **(D)** Circle diagram of GO enrichment analysis.

For each disease dataset, we independently integrated genes from three screening methods: WGCNA-derived module genes, DEGs, and LASSO-selected signature genes, and merged them into a single disease-associated gene list after removing duplicates. Specifically, the DED-associated list (GSE44101) comprised genes from the darkolivegreen4 module (1,283 genes), DEGs (5,939 genes), and LASSO-selected genes (7 genes). The anxiety-associated list (GSE98793) comprised genes from the green module (1,106 genes), DEGs (135 genes), and LASSO-selected genes (7 genes). The intersection of these two disease-associated lists yielded 128 candidate genes shared between DED and anxiety (**Figure 4C**). Subsequent enrichment analysis was conducted using R to elucidate the functions of these screened genes and their involvement in signaling pathways. The findings revealed that the identified genes were predominantly enriched in pathways related to Circadian rhythm, Hematopoietic cell lineage, Platelet activation, Lysosome, Pyrimidine metabolism, and Long-term potentiation. The primary functions of these genes were associated with microtubule nucleation, microtubule polymerization or depolymerization, chromosome segregation within biological processes, spindle microtubule, microtubule plus-end, and chromosomal regions within cellular components. Additionally, they exhibited molecular functions such as mannosyl-oligosaccharide mannosidase activity, poly(A)-specific ribonuclease activity, and alpha-mannosidase activity, as illustrated in **Figure 5**.

#### 3.1.5 PPI network construction and modular analysis

**Figure 6:**
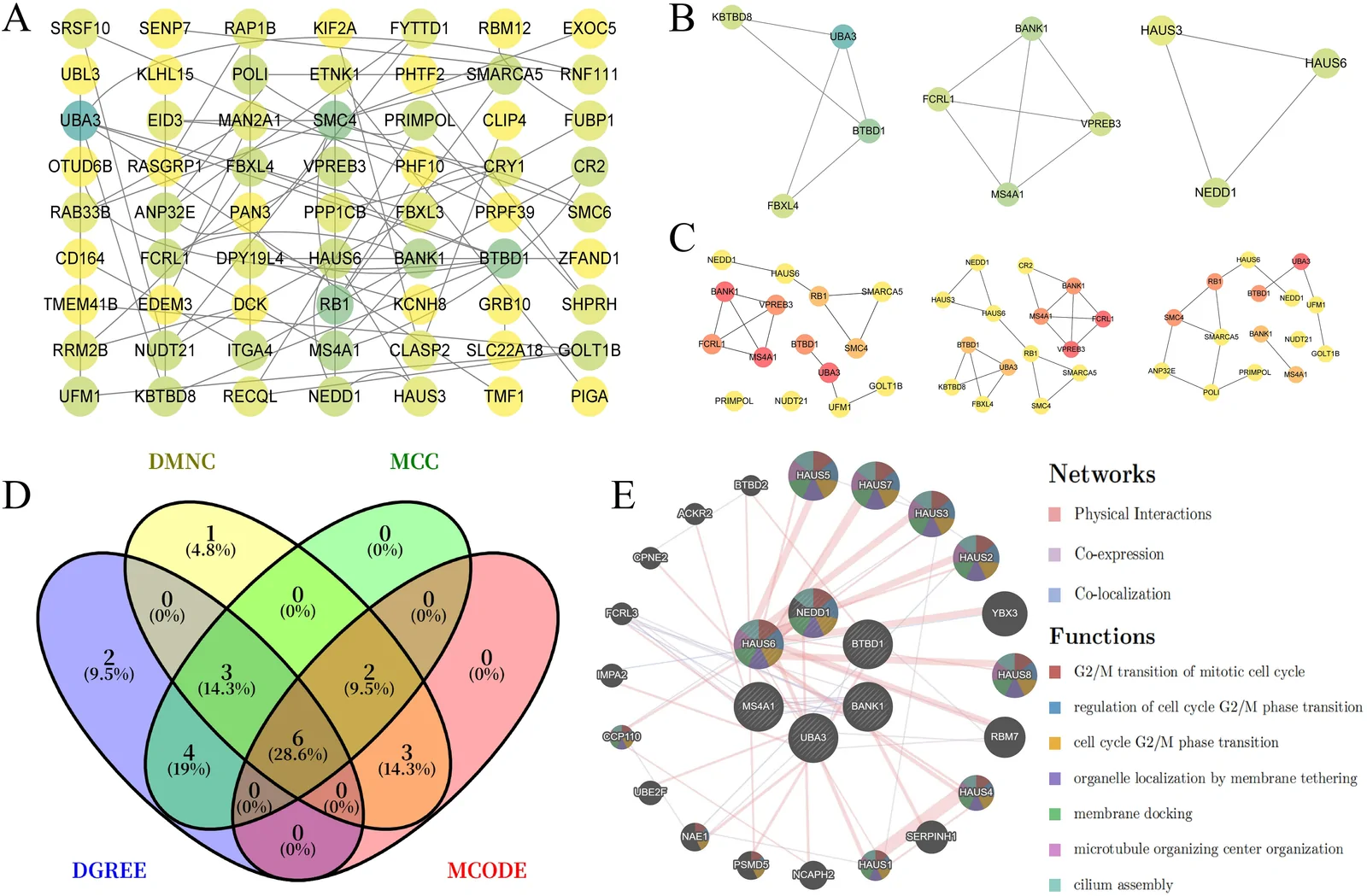
PPI network construction and modular analysis **(A)** PPI networks from String10.0. Yellow to green indicates higher degree values. **(B)** Clusters 1, 2 and 3 from the MCODE analysis of candidate shared genes. **(C)** Identification of candidate shared genes using CytoHubba of MCC, DMNC, and Degree ranking methods. **(D)** Identifcation of hub genes by CytoHubba and MCODE method. **(E)** Hub genes and their co-expression genes were analyzed by GeneMANIA.

The genes identified through WGCNA, Differentially Expressed Genes, and machine learning analysis were imported into the STRING database (v10.0, available at https://string-db.org/), with the species parameter set to “Homo sapiens” to identify genes comorbid in DED and anxiety. These genes underwent PPI analysis, and the key comorbid genes were identified, as depicted in **Figure 6A**. Utilizing the MCODE plugin, three distinct clustering modules of closely interconnected genes were extracted (**Figures 6B, C**). Cluster 1 comprised 4 nodes and 5 edges, Cluster 2 comprised 4 nodes and 6 edges, and Cluster 3 comprised 3 nodes and 3 edges.

#### 3.1.6 Selection and analysis of hub genes

In addition, network analysis was conducted utilizing the CytoHubba plugin, employing MCC, DMNC, and Degree values as reference standards, which led to the identification of 15 key genes. Subsequently, we integrated the results from MCC, DMNC, Degree, and MCODE analyses to isolate six hub genes: *UBA3*, *BTBD1*, *MS4A1*, *BANK1*, *HAUS6*, and *NEDD1*, as shown in **Figure 6D**. Following this, we assessed the co-expression network and the functional roles of these hub genes using the GeneMANIA database. The network analysis revealed that 34.90% of the interactions were co-expression, 63.18% were physical interactions, and 1.93% were co-localization, as illustrated in **Figure 6G**, as shown in **Figure 6E**.

### 3.2 Validation

**Figure 7:**
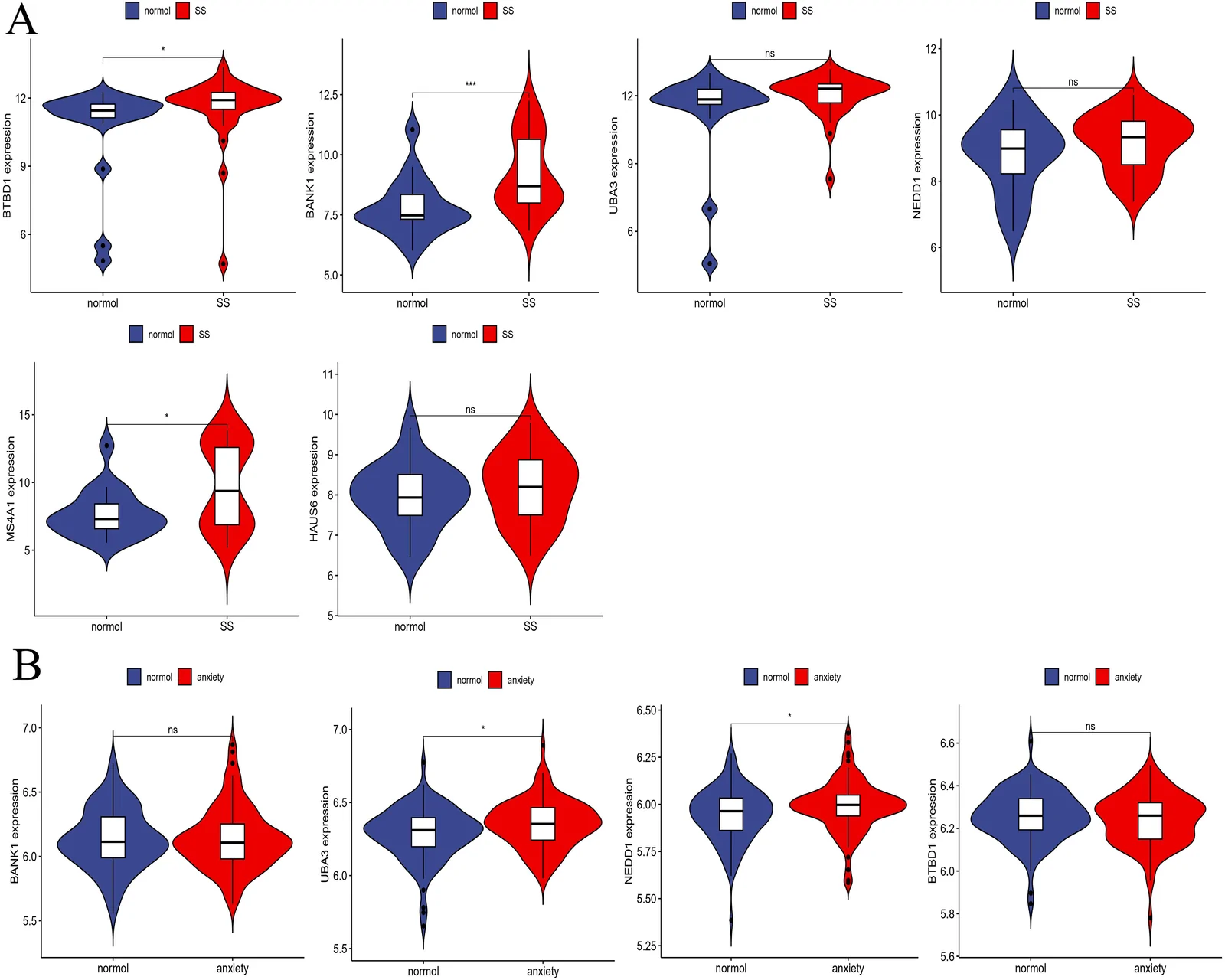
Validation of hub genes expression. **(A)** By DED dataset (GSE40611). **(B)** By anxiety datasets (GSE61672). Data are presented as mean ± SD. *P < 0.05, ***P < 0.001, vs. Control group. *n* = 18 (normal) and 31 (disease) for GSE40611; *n* = 179 (normal) and 157 (disease) for GSE61672.

The expression levels of six key genes were validated using the anxiety-related dataset GSE61672 and the DED-related dataset GSE40611, with a significance threshold set at *P* < 0.05. Our analysis revealed that the expression levels of *BTBD1*, *MS4A1*, and *BANK1* were significantly elevated (*P* < 0.05) in an additional DED-related dataset (**Figure 7A**). Similarly, *UBA3* and *NEDD1* expression levels were significantly elevated (*P* < 0.05) in an alternative anxiety-related dataset (**Figure 7B**). Based on these findings, we propose the hypothesis that *BTBD1*, *MS4A1*, *BANK1*, *UBA3*, and *NEDD1* may exhibit comorbidity in anxiety and DED.

### 3.3 Immune infiltrate analysis

**Figure 8:**
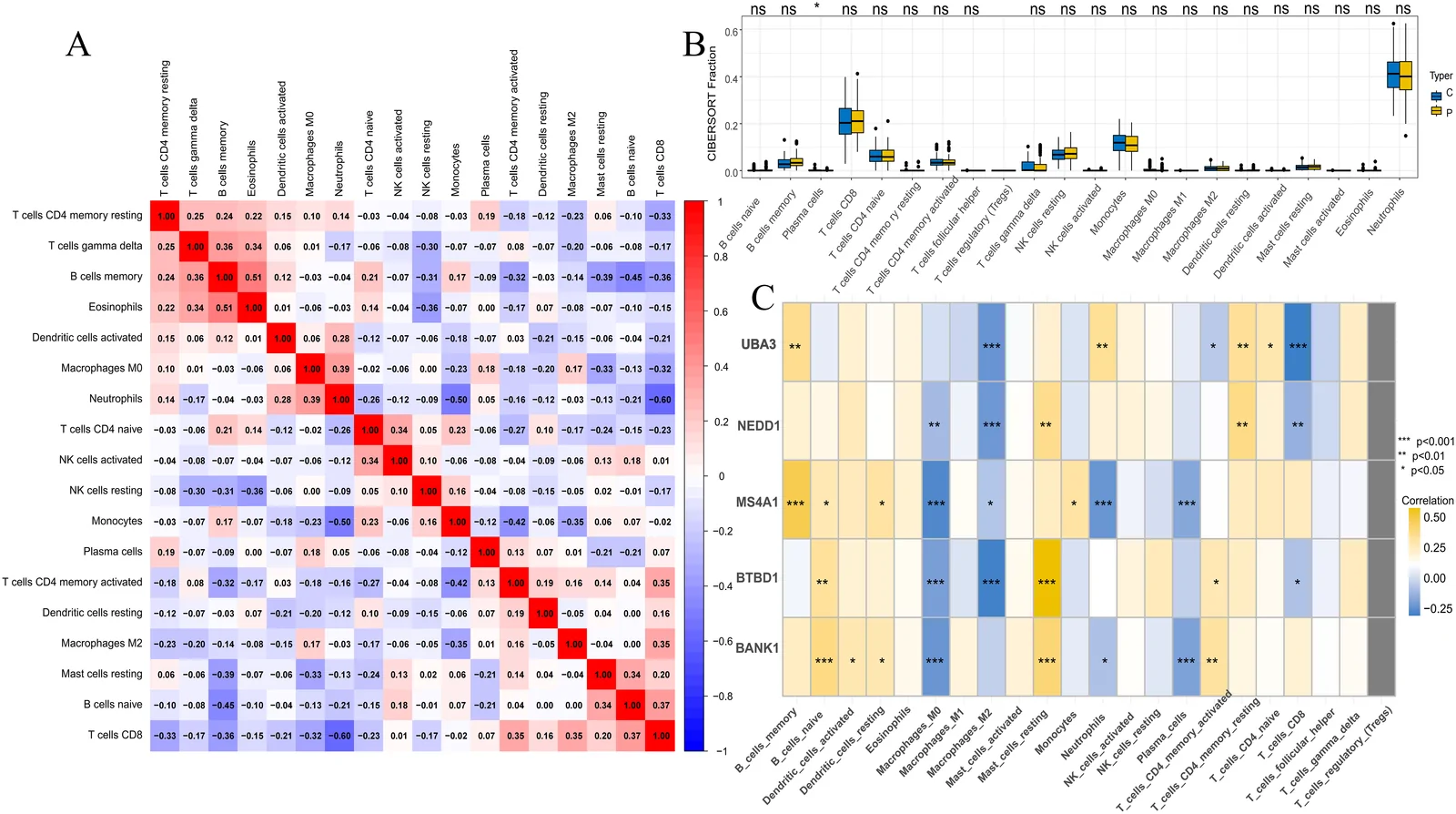
Immune infiltration analyses. **(A)** Heatmap of correlation analysis of different immune cell infiltration. **(B)** Box plot of correlation analysis of different immune cell infiltration. **(C)** Heatmap of correlation between hub genes and immune infiltrations. *p<0.05, **p<0.01, ***p<0.001.

**Figure 9:**
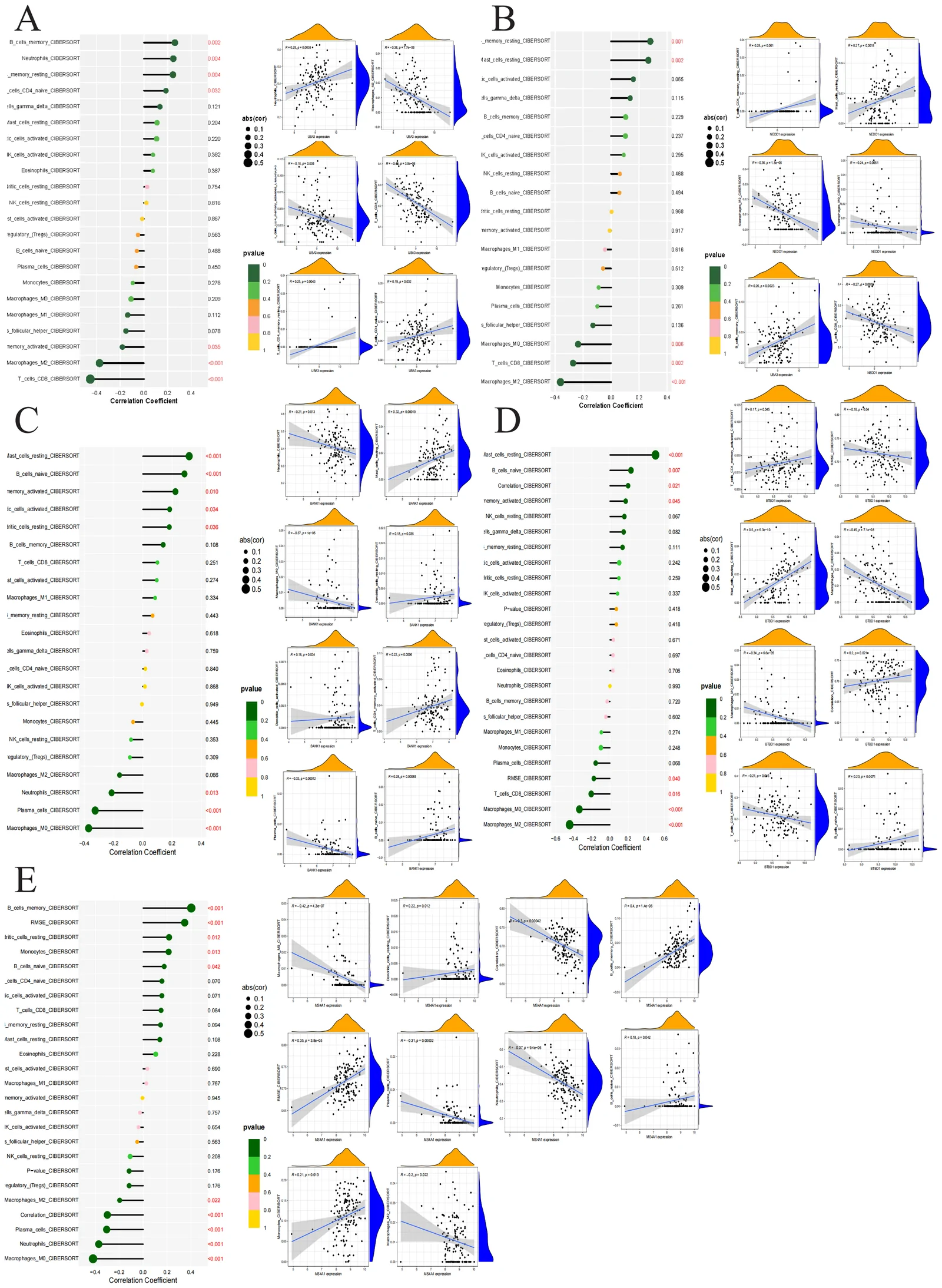
The correlation between hub genes expression and immune infiltration. **(A)** *UBA3*. **(B)** *NEDD1*. **(C)** *BANK1*. **(D)** *BTBD1*. **(E)** *MS4A1*.

The CIBERSORT analysis demonstrated that, based on datasets related to DED and anxiety, there is a correlation between the comorbidity of anxiety and DED and the immune responses of plasma cells (**Figure 8**). The findings concerning the association between characteristic genes and immune cell infiltration revealed that *BTBD1* is involved in naive B cells, M0 and M2 macrophages, resting mast cells, activated CD4 memory T cells, and CD8 T cells. *MS4A1* is associated with memory and naive B cells, resting dendritic cells, M0 and M2 macrophages, monocytes, neutrophils, and plasma cells. *BANK1* is linked to naive B cells, activated and resting dendritic cells, M0 macrophages, resting mast cells, neutrophils, plasma cells, and activated CD4 memory T cells. *UBA3* is involved in memory B cells, M2 macrophages, neutrophils, activated and resting CD4 memory T cells, naive CD4 T cells, and CD8 T cells. *NEDD1* is associated with M0 and M2 macrophages, resting mast cells, resting CD4 memory T cells, and CD8 T cells (**Figure 9**).

### 3.4 The single-cell RNA sequencing analysis

**Figure 10:**
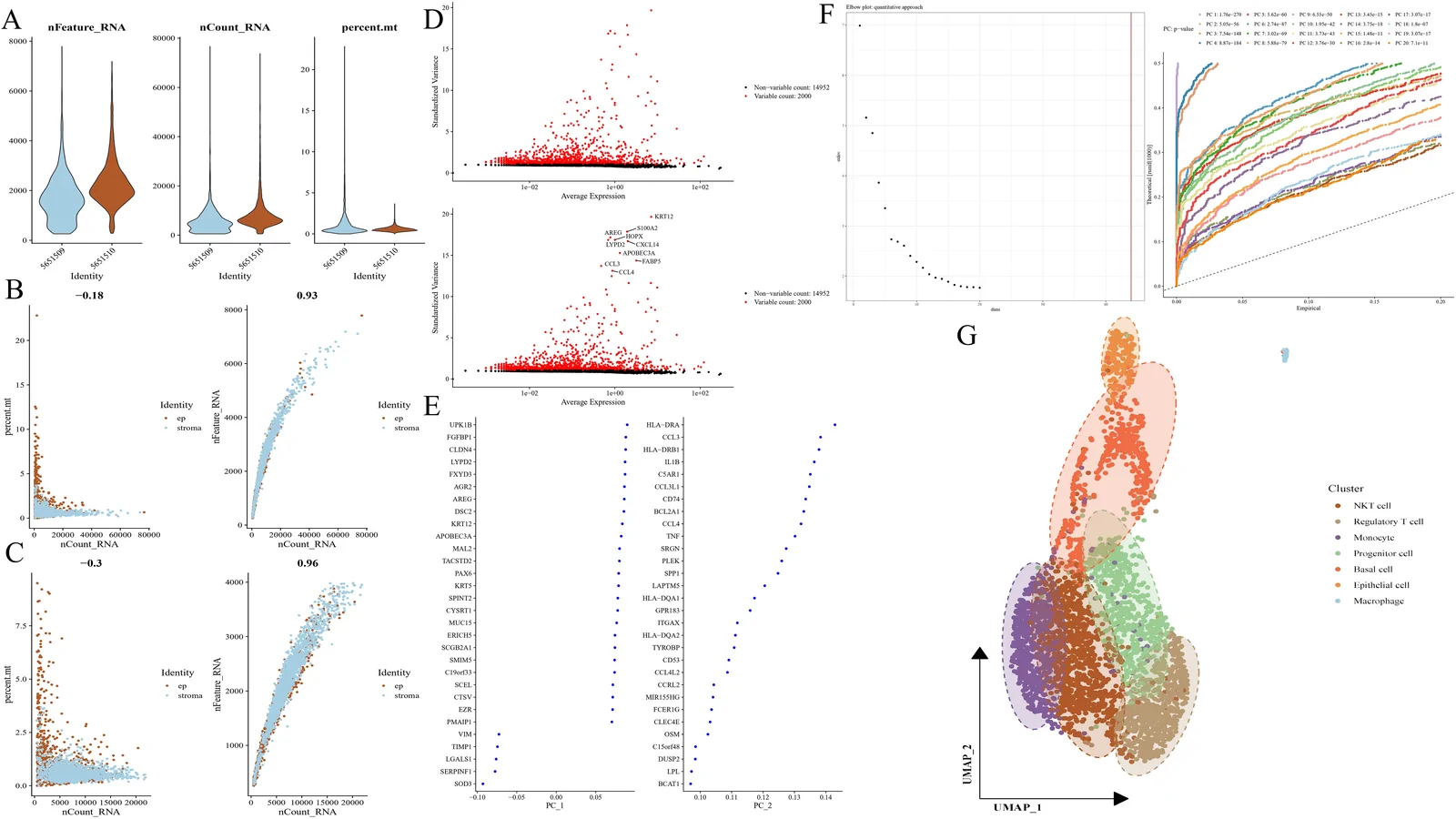
Single-cell RNA sequencing analysis **(A)** Gene data in the cell. **(B)** Correlation between basic data before filtering. **(C)** Correlation between filtered base data. **(D, E)** The screening of the hypervariable genes. **(F)** The ElbowPlot function was used to evaluate PC and visualization results. **(G)** Proportional distribution of cells in the sample by UMAP method.

For further analysis, single-cell RNA sequencing data from corneal epithelial and stromal tissues, obtained from GSE186433 and generated using the GPL24676 platform, were utilized. Initially, quality control procedures were applied to the cellular data, standardizing RNA counts between 200 and 4,000. Cells with elevated mitochondrial content (>20%) were excluded from the analysis (**Figure 10A**). Subsequently, batch effect correction was performed on the filtered dataset using a subset of highly variable genes, identified as those exhibiting the most significant expression variability across cells (**Figures 10B, C**). These highly variable genes were then subjected to principal component analysis for dimensionality reduction (**Figures 10D, E**). Following cell annotation, six distinct cell types were identified within the corneal epithelial and stromal tissues: Macrophage, Epithelial cell, Basal cell, Progenitor cell, Monocyte, Regulatory T cell, and NKT cell (**Figures 10F, G**).

**Figure 11:**
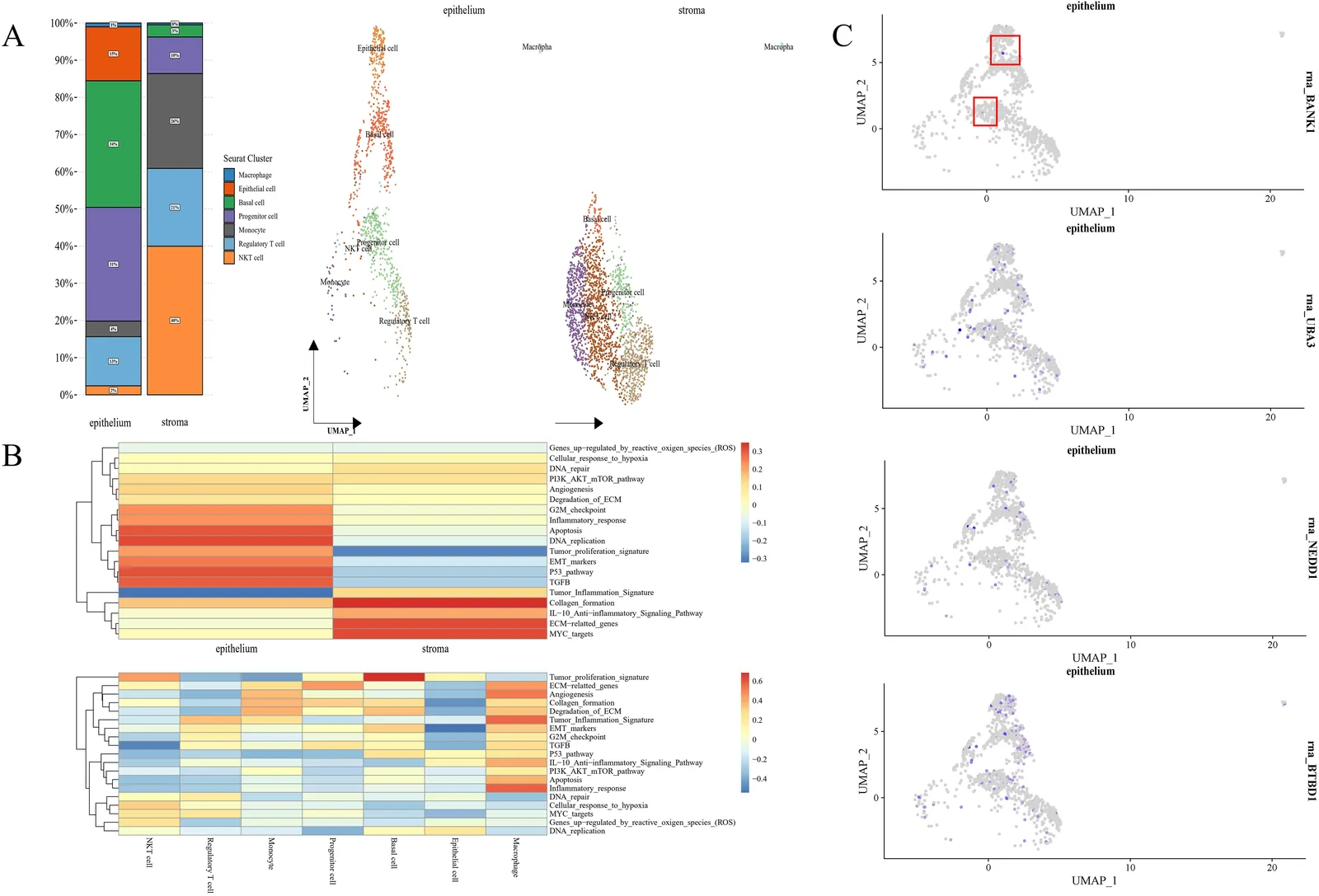
**(A)** Proportional histogram analysis. **(B)** Metabolic analyses. **(C)** The single-cell RNA sequencing analysis of *BANK1*, *UBA3*, *NEDD1* and *BTBD1*.

Proportional histogram analysis revealed differences in cell type enrichment between the corneal epithelium and stroma. Progenitor cells were most enriched in the epithelium, while natural killer T (NKT) cells were predominant in the stroma, and macrophages were more common in the epithelium (**Figure 11A**). Metabolic analysis showed distinct biological processes for each cell type, with the epithelium primarily linked to inflammatory response, apoptosis, and DNA replication (**Figure 11B**). Expression analysis of *BTBD1*, *MS4A1*, *BANK1*, *UBA3*, and *NEDD1* in the epithelium revealed that *BTBD1*, *BANK1*, *UBA3*, and *NEDD1* were expressed across various cell types (**Figure 11C**). We further evaluated whether these hub genes were associated with DED-anxiety comorbidity *in vivo.*

### 3.5 *In vivo* experiment validation

#### 3.5.1 Pathological and behavioral phenotypes

**Figure 12:**
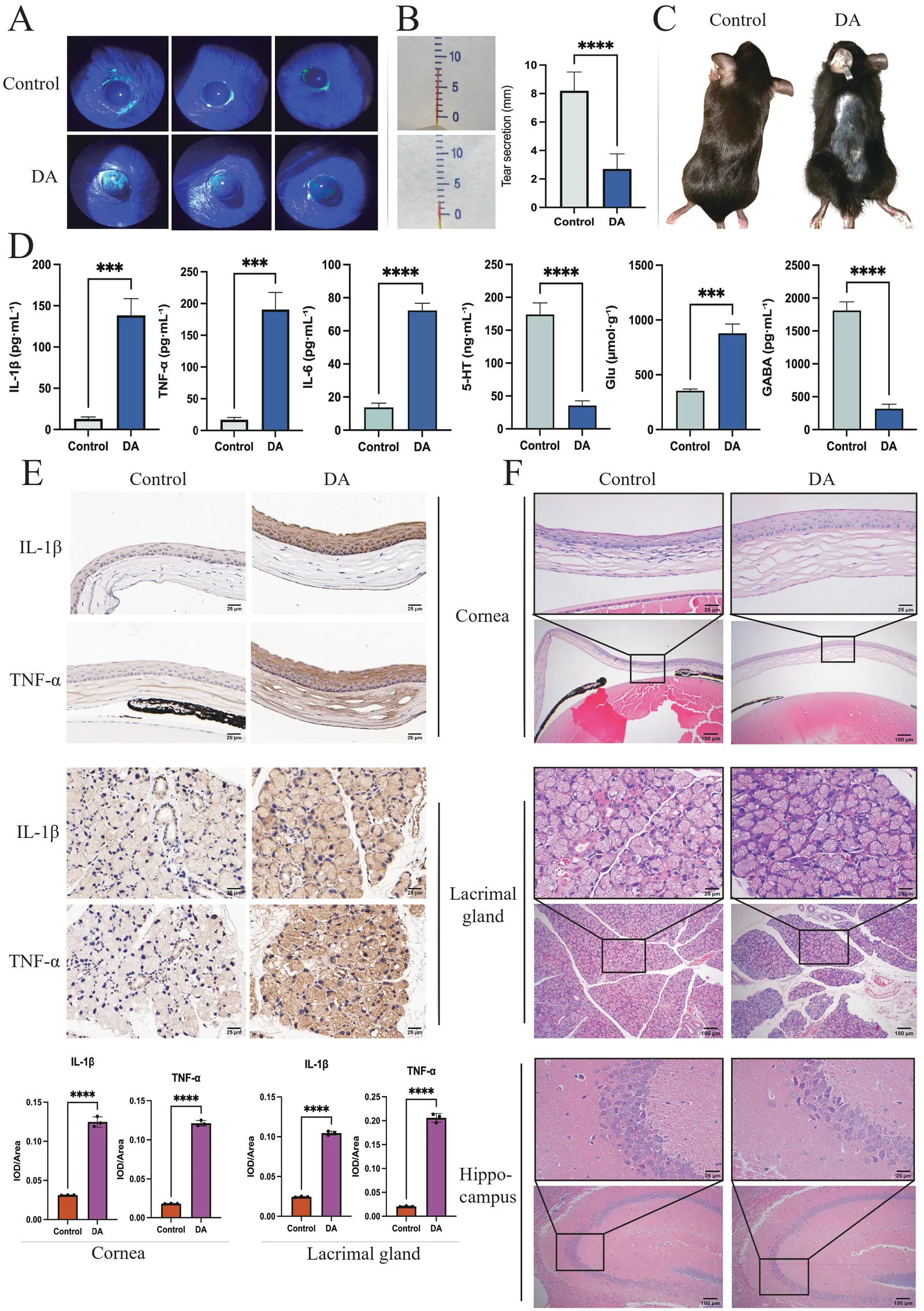
Ocular surface parameters, systemic inflammation, and hippocampal neurotransmitter levels in the comorbidity of DED-anxiety mouse model. **(A)** Representative corneal fluorescein staining images. **(B)** Tear secretion measured by phenol red thread test. **(C)** Representative images showing over-grooming behavior in DA-model mice compared with controls. **(D)** Levels of IL-1β, TNF-α, and IL-6, 5-HT, GABA, and Glu measured by ELISA. The highlight should be as follows: **(E)** Representative IHC staining of IL-1β and TNF-α in corneal tissue. Scale bar = 25 mm for IHC images. **(F)** Representative H&E staining of lacrimal gland, cornea, and hippocampus. Data are presented as mean ± SD. ***P < 0.001, ****P < 0.0001 vs. Control group; ns, not significant. *n* = 6 per group for ELISA and tear secretion; *n* = 3 per group for IHC.

The corneal barrier function was evaluated using fluorescein staining, while tear production was assessed through the phenol red thread test. In comparison to the control group, the model mice demonstrated significant central corneal staining and a substantial reduction in tear secretion. Additionally, the model group exhibited pronounced over-grooming behavior, suggestive of an anxiety-like phenotype. Systemic inflammation was quantified using ELISA, revealing significantly elevated serum levels of IL-1β, TNF-α, and IL-6 in the model group. In the hippocampus, the DA group displayed a notable neurotransmitter imbalance, characterized by significantly decreased levels of 5-HT and GABA, alongside markedly increased Glu levels compared to the control group. IHC staining of corneal and lacrimal tissues corroborated the heightened activity of inflammatory cytokines, with model mice exhibiting strong immunoreactivity for IL-1β (*P* < 0.0001) and TNF-α (*P* < 0.0001). H&E staining was conducted to investigate tissue remodeling and local inflammatory infiltration. In model mice, there was observed thinning of the corneal epithelium, disorganization of the stroma, and acinar damage within the lacrimal gland. In the hippocampus, neuronal loss and disordered arrangement were evident, as shown in **Figure 12**.

**Figure 13:**
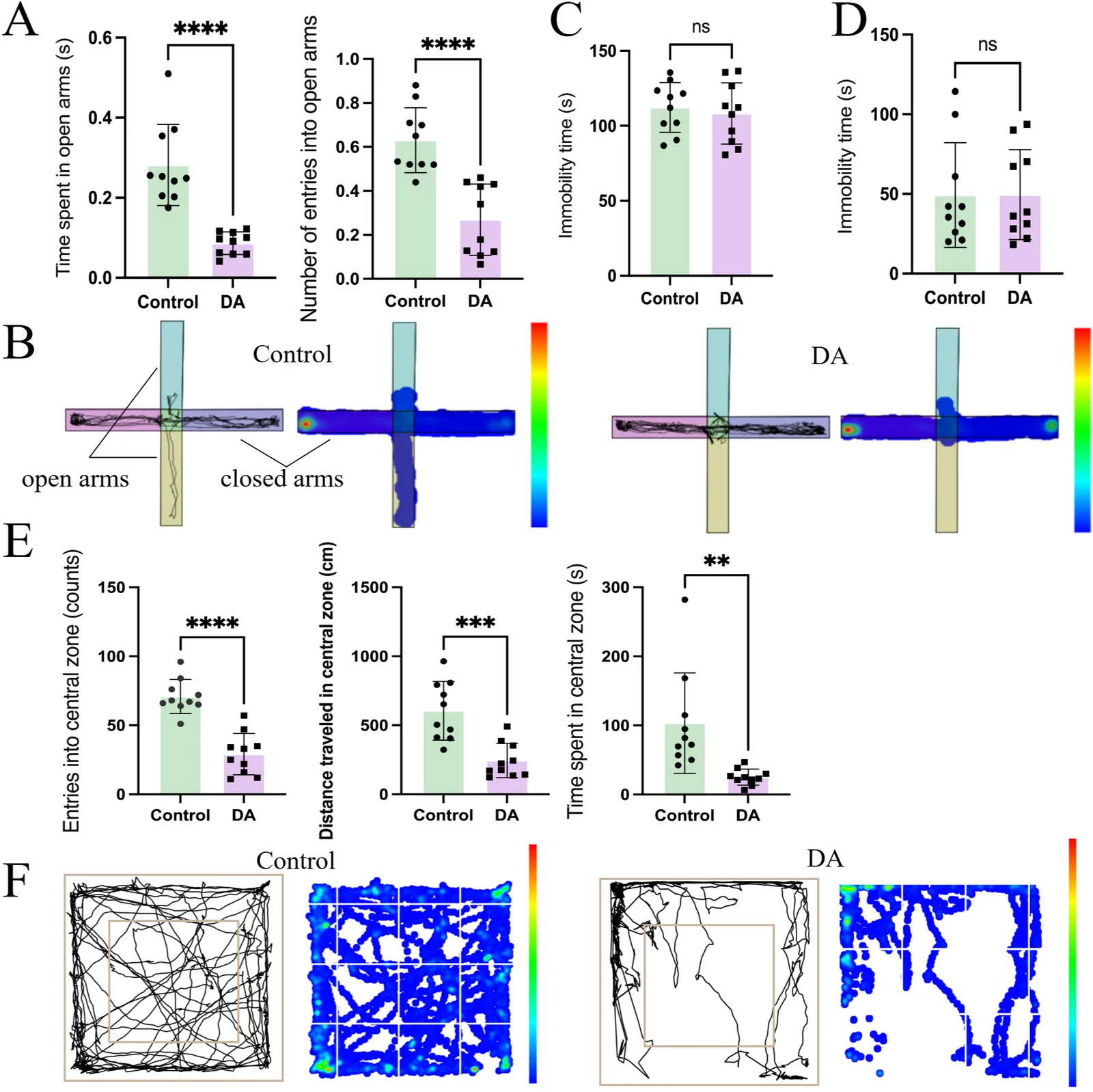
Behavioral assessments of anxiety-like and depressive-like behaviors in the comorbidity of DED-anxiety mouse model. **(A)** Time spent in open arms and number of entries into open arms in the EPM test. **(B)** Representative trajectory maps of mice in the EPM, with open arms and closed arms indicated. **(C)** Immobility time in the TST. **(D)** Immobility time in the FST. **(E)** Number of entries into the central zone, distance traveled in the central zone, and time spent in the central zone in the OFT. **(F)** Representative trajectory maps of mice in the OFT. Data are presented as mean ± SD. **P < 0.01, ***P < 0.001, ****P < 0.0001 vs. Control group; ns, not significant. *n* = 10 per group.

During the open field test, DA-model mice exhibited increased anxiety-like behavior, as evidenced by spending less time in the central zone, traveling a shorter distance in the center, and entering the center fewer times compared to the control group. Similarly, in the elevated plus maze, DA-model mice demonstrated fewer entries into the open arms and a reduced proportion of time spent in the open arms relative to controls, further indicating an anxiety-like phenotype. Conversely, there were no significant differences between the groups in immobility time during the forced swim and tail suspension tests, suggesting an absence of pronounced depressive-like behavior. These findings confirm the successful establishment of a comorbidity of DA-model, which was subsequently employed to validate bioinformatically predicted immune mechanisms, as shown in **Figure 13**. These behavioral and pathological alterations provided the phenotypic foundation for subsequent multi-tissue gene expression analysis, enabling us to examine whether the candidate hub genes identified through bioinformatics correlated with the observed inflammatory and neurobiological changes.

#### 3.5.2 Multi-tissue gene expression profiling

**Figure 14:**
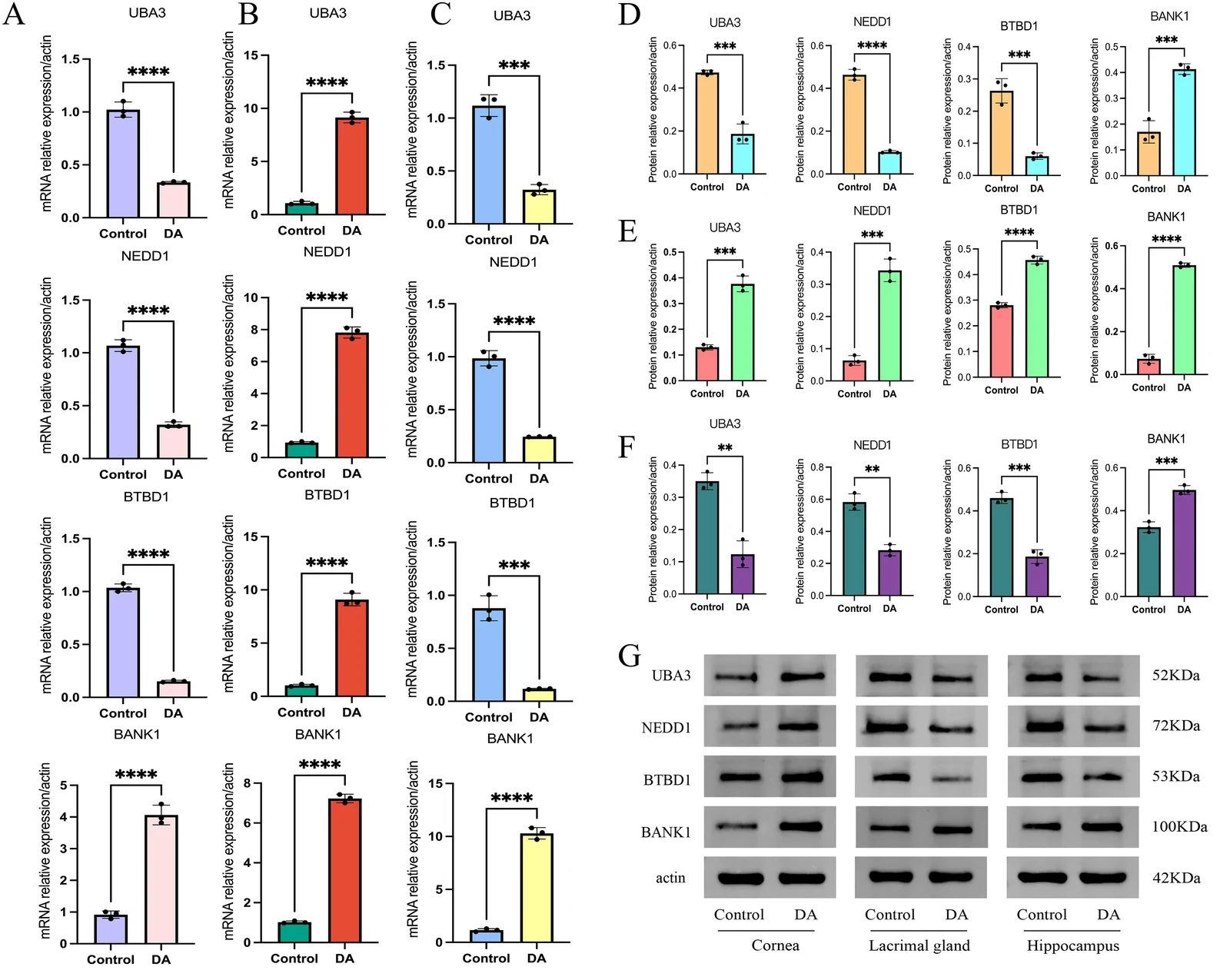
Tissue-specific expression validation of hub genes in the comorbidity of DED-anxiety mouse model. **(A–C)** Relative mRNA expression levels of *UBA3*, *NEDD1*, *BTBD1*, and *BANK1* in the lacrimal gland **(A)**, cornea **(B)**, and hippocampus **(C)** measured by RT-qPCR. **(D-F)** Relative protein expression levels of *UBA3*, *NEDD1*, *BTBD1*, and *BANK1* in the lacrimal gland **(D)**, cornea **(E)**, and hippocampus **(F)** measured by Western blot. **(G)** Representative blot images shown above each quantification panel. β-actin was used as the loading control. Data are presented as mean ± SD. **P < 0.01, ***P < 0.001, ****P < 0.0001 vs. Control group; ns, not significant. *n* = 3 per group.

In the corneal tissue, the mRNA expression levels of *UBA3* (*P* < 0.0001), *NEDD1* (*P* < 0.0001), *BTBD1*(*P* < 0.0001), *BANK1* (*P* < 0.0001) were significantly upregulated compared to the control group. In the lacrimal gland, *BANK1* (*P* < 0.0001) expression was markedly increased, whereas *BTBD1* (*P* < 0.0001), *UBA3* (*P* < 0.0001), *NEDD1* (*P* < 0.0001) were significantly downregulated. In the hippocampus, a similar trend to the lacrimal gland was observed, with elevated *BANK1* (*P* < 0.0001) expression and reduced expression of *BTBD1* (*P* < 0.001), *UBA3* (*P* < 0.001), *NEDD1* (*P* < 0.0001). Consistent with the RT-qPCR findings, WB results revealed: Significant upregulation of BTBD1 (*P* < 0.0001), BANK1 (*P* < 0.0001), UBA3 (*P* < 0.001), NEDD1 (*P* < 0.001) protein levels in corneal tissues, indicating activation of local immune and stress-response pathways. In contrast, in the lacrimal gland and hippocampus, BANK1 (*P* < 0.001) protein expression was significantly increased, whereas BTBD1 (*P* < 0.001), UBA3 (*P* < 0.001 or *P* < 0.01), NEDD1 (*P* < 0.0001 or *P* < 0.01) were markedly decreased, as shown in **Figure 14**.

## 4 Discussion

DED is a multifactorial disorder of the ocular surface, characterized by tear film instability, elevated tear osmolarity, and chronic inflammation, which collectively result in ocular discomfort, visual disturbances, and damage to the ocular surface (31, 32). In addition to its localized effects, DED is increasingly acknowledged as a systemic condition with intricate neuroendocrine and immune components (33). Conversely, anxiety disorders are among the most common psychiatric conditions, characterized by excessive worry, autonomic hyperarousal, and significant impairment in quality of life (34). Previous epidemiological studies and meta-analyses have reported an association between DED and anxiety symptoms (35, 36). Emerging evidence points to shared neuroimmune mechanisms linking DED and psychiatric disorders, including hypothalamic-pituitary-adrenal axis dysregulation, systemic and ocular inflammation, and impaired neurotrophic signaling (37). The ubiquitin-like modifier NEDD8 and its neddylation pathway have also been implicated in autoimmune and inflammatory processes relevant to both conditions (38). This has led to a growing recognition of DED and anxiety as comorbid conditions rather than isolated phenomena. From an immunological standpoint, both DED and anxiety are associated with dysregulated inflammatory responses. In DED, the activation of both innate and adaptive immune pathways, characterized by the upregulation of pro-inflammatory cytokines, chemokines, and T cell–mediated responses on the ocular surface, is pivotal in the onset and persistence of the condition (39, 40). Similarly, anxiety disorders have been associated with systemic low-grade inflammation and altered peripheral cytokine profiles, while dysregulation of the hypothalamic–pituitary–adrenal axis and autonomic nervous system may also contribute to their neuroimmune pathophysiology (41–43). These findings suggest that shared immune-related mechanisms may partially account for the bidirectional relationship between ocular surface pathology and psychological distress. Nonetheless, current research on the comorbidity of DED and anxiety is predominantly based on cross-sectional surveys and symptom-based questionnaires, which are characterized by heterogeneous diagnostic criteria and a lack of mechanistic investigation. There is a scarcity of studies that directly examine immune mediators, immune cell signatures, or comorbid gene networks specific to individuals with both DED and anxiety. Consequently, the temporal and causal relationships between immune dysregulation, ocular surface damage, and anxiety remain largely unresolved. The evident gap in the existing literature highlights the necessity for a comprehensive approach that transcends descriptive epidemiology to thoroughly analyze the immune and molecular characteristics of the comorbidity between DED and anxiety. In this regard, our study aims to identify common immune-related pathways and comorbid gene signatures associated with DED and anxiety. Our objective is to elucidate their potential pathogenic interactions and to establish a theoretical foundation for the development of more precise, immune-targeted future mechanistic studies and biomarker exploration for patients afflicted with both conditions.

In the current investigation, employing a comprehensive bioinformatics pipeline that integrates WGCNA, differential expression analysis, and LASSO regression, we identified 128 candidate genes potentially linked to the comorbidity of DED and anxiety. Functional enrichment analysis indicated that these genes are predominantly involved in pathways including circadian rhythm, hematopoietic cell lineage, platelet activation, lysosome, and long-term potentiation. Among these pathways, circadian rhythm emerged as a noteworthy signal in the enrichment analysis. These findings provide a plausible biological context for the enrichment of circadian rhythm-related pathways in our analysis. Specifically, core clock genes modulate the expression of pro-inflammatory cytokines and the trafficking of immune cells, while in the ocular context, circadian disruptions can alter tear film composition and exacerbate inflammation of the ocular surface (44), Circadian rhythm disruption can intensify inflammatory responses by affecting the balance of the ocular surface microbiota. Research has demonstrated that such disruptions lead to dysregulation of clock genes in the conjunctiva and an upregulation of pro-inflammatory mediators, including TNF-α, IL-6, and IL-17. These modifications activate IL-17-mediated inflammatory pathways within conjunctival tissues, thereby exacerbating inflammation of the ocular surface (45). Chronic jet lag disrupts circadian organization in the lacrimal gland and induces glandular hyperproliferation accompanied by reactive oxygen species accumulation (46). Moreover, the deletion of core clock genes leads to the loss of circadian rhythms in tear secretion, further substantiating the essential role of clock genes in regulating tear secretion (47). Taken together, the enrichment of circadian rhythm pathways in our comorbid gene set may reflect a shared biological interface connecting peripheral ocular inflammation with central neuroimmune dysregulation. In the ocular surface, circadian disruption promotes inflammatory cytokine production and compromises epithelial barrier function; in the central nervous system, it dysregulates HPA axis activity and monoaminergic neurotransmission. Nevertheless, the circadian rhythm findings in this study should be interpreted with caution. Although bioinformatics analysis suggested the involvement of circadian-related pathways, our experimental validation did not directly assess circadian oscillations, clock gene rhythmicity, light-dark cycle responses, or time-dependent changes in ocular and behavioral phenotypes. Therefore, the present results support a potential association between circadian rhythm-related molecular processes and DED-anxiety comorbidity.

Through a series of analyses, we identified *BTBD1*, *BANK1*, *UBA3*, and *NEDD1* as potential hub genes, with their differential expression confirmed in our comorbidity mouse model. BANK1, an adaptor protein predominantly expressed in B cells, serves as a critical regulator of B-cell receptor (BCR) signaling and innate immune responses (48). Its upregulation in our model is consistent with the growing acknowledgment of B-cell involvement in both autoimmune DED and stress-induced immune dysregulation. *UBA3* is the catalytic subunit of the NEDD8-activating enzyme and plays an important role in protein neddylation, a post-translational modification closely associated with the pathogenesis of various inflammatory diseases (49). Recent evidence indicates that NEDD8-dependent modification stabilizes NLRP3 and enhances inflammasome activation, and pharmacological inhibition of this pathway alleviates stress-induced NLRP3 inflammasome activation and anxiety-like behaviors in mice (50). The neddylation pathway in the ventral hippocampus is activated by chronic stress, and its inhibition reverses anxiety-and depressive-like behaviors (51). Together, these observations support the hypothesis that *UBA3* may contribute to anxiety-related behavioral changes through its modulation of NLRP3 inflammasome activity and neuroinflammatory pathways. In murine models, myeloid cells deficient in UBA3 showed resistance to chemically induced colitis and exhibited diminished anxiety-like behaviors under psychological stress.

The experimental validation via RT-qPCR and Western blotting provided further insights into the tissue-specific regulatory landscape of these hub genes. In the corneal tissue, all four hub genes (*BTBD1*, *BANK1*, *UBA3*, and *NEDD1*) exhibited consistent and significant upregulation at both mRNA and protein levels, corroborating the bioinformatic prediction that local ocular surface inflammation was accompanied by altered expression of these immune-related and stress-response genes. However, divergent expression patterns emerged in the lacrimal gland and hippocampus. While *BANK1* expression remained markedly elevated across all three tissues, *BTBD1*, *UBA3*, and *NEDD1* were significantly downregulated in the lacrimal gland and hippocampus compared to the cornea. This tissue-specific dichotomy may reflect distinct functional roles of these genes in different anatomical compartments. The persistent upregulation of *BANK1* across all tissues underscores its potential association in B-cell-mediated immune responses that may be associated with systemic stress signaling with local tissue pathology. Conversely, the downregulation of *BTBD1*, *UBA3*, and *NEDD1* in the lacrimal gland and hippocampus may indicate compensatory or maladaptive responses to chronic stress and desiccating injury, potentially contributing to lacrimal gland dysfunction and altered neuroplasticity associated with anxiety. Notably, the hippocampal downregulation of UBA3 observed in our model warrants careful interpretation. Several factors may account for this expression pattern. First, differences in stress paradigms, hippocampal subregions, sampling time points, and cell-type composition may lead to distinct expression profiles. Second, UBA3 expression alone may not directly reflect global neddylation activity, which is also influenced by other components of the NEDD8 conjugation machinery, substrate availability, and deneddylation processes. Alternatively, reduced UBA3 expression may represent a compensatory negative-feedback response to earlier or cell-specific activation of neddylation. However, because global NEDD8 conjugation and the neddylation status of downstream substrates were not directly measured in the present study, these explanations remain speculative and require validation through time-course, cell-specific, and functional experiments. These findings collectively suggest that the comorbidity of DED and anxiety is not driven by a uniform transcriptional program across all affected tissues, but rather by a complex, tissue-specific interplay of shared immune-inflammatory pathways. These tissue-specific molecular changes, together with the B-cell/plasma cell infiltration patterns observed in our immune analysis and the hippocampal neurotransmitter imbalance associated with anxiety-like behaviors, collectively point to a model in which shared immune-inflammatory pathways may bridge peripheral ocular surface inflammation with central neurobiological alterations.

Our analysis of immune infiltration further substantiates the immunological link between DED and anxiety. Utilizing CIBERSORTx deconvolution, we identified a significant correlation between the comorbidity signature and the infiltration abundance of plasma cells and naïve B cells. Notably, the hub gene BANK1 showed exploratory correlations with B cell lineages and plasma cells. *BANK1* is a scaffold protein that regulates B-cell receptor and Toll-like receptor signaling, and its deficiency alters B-cell differentiation and plasma cell formation (52). Moreover, *BANK1* deficiency reshapes the gut microbiota toward an anti-inflammatory composition and reduces lupus-associated gut pathology (53). This finding aligns with the pathophysiology of DED, which is characterized by ocular surface inflammation involving immune cell infiltration and, in severe cases, autoantibody production (54). To achieve enhanced spatial and cellular resolution, we conducted an analysis of single-cell RNA sequencing datasets from corneal tissue. The exploratory correlation analyses suggested that the hub genes *BTBD1*, *BANK1*, *UBA3*, and *NEDD1* were not confined to a single cell type but were broadly expressed across various populations within the corneal epithelium, including epithelial cells, progenitor cells, and notably, macrophages. The pronounced presence of macrophages in the corneal epithelium is consistent with their role as key components of the innate immune response at the ocular surface (55, 56). The concurrent expression of these hub genes within both the structural (epithelial) and immune (macrophage) compartments of the cornea indicates a model in which systemic stress or anxiety signals may prime or activate these shared pathways within local tissue environments. This activation could potentially lower the threshold for ocular surface inflammation or compromise epithelial repair mechanisms. Conversely, persistent ocular surface inflammation may upregulate the expression of these immune-related genes, thereby contributing to the neuroinflammatory environment associated with anxiety. These findings suggest that resident and infiltrating cells of the ocular surface may participate in molecular processes associated with DED–anxiety comorbidity, providing a cellular context for future investigation of the potential bidirectional interplay between ocular surface inflammation and anxiety-related neuroimmune alterations. However, it should be noted that these immune correlation findings are exploratory in nature, as they were derived from raw *P*-values without multiple-testing correction, and therefore warrant validation in independent cohorts.

The DED model, based on BAC and desiccation stress, in conjunction with CUMS, offers a relatively comprehensive comorbidity framework that more accurately mirrors clinical situations where ocular surface damage coexists with chronic psychosocial stress. By concurrently inducing tear film instability, corneal epithelial damage, and anxiety-like behaviors, this model facilitates the simultaneous examination of ocular and neurobehavioral phenotypes. The application of multiple, complementary assessment methods, including tear secretion measurement, corneal fluorescein staining, histopathological analysis, immunohistochemistry, Western blotting, and RT-qPCR for the ocular surface and lacrimal gland, alongside several standardized behavioral tests, which enhances both construct and face validity, thereby enabling mechanistic investigations at tissue and molecular levels. The aim of the present study was to address this gap by employing an innovative methodology. Our analysis was enhanced through the integration of extensive Anxiety and DED datasets sourced from a continuously updated GEO database. The predictive model developed for comorbidity offers a theoretical framework that paves the way for future research on metabolic processes and the therapeutic modulation of metabolic disturbances associated with Anxiety and DED. The novelty of comorbidity research lies in its shift from viewing diseases as isolated entities to highlighting their interrelations and interactions. Within this context, DED and anxiety are no longer seen as separate clinical issues but as interrelated conditions within shared neuroendocrine and immune networks. Investigating their comorbidity facilitates a more holistic understanding of the interaction between ocular surface pathology and affective dysregulation, thereby offering more comprehensive guidance for diagnosis, risk stratification, and therapeutic decision-making. By concurrently examining a range of biological and environmental factors, research focused on comorbidity is more adept at capturing the complexity and diversity of disease manifestations, thereby informing more effective prevention and treatment strategies. In this context, the novelty of the present study lies primarily in its application of advanced and multifaceted bioinformatics techniques to systematically elucidate the association and interaction landscape between DED and anxiety. Through integrated transcriptomic and systems-level analyses, we identified candidate genes, pathways, and immune signatures potentially shared by DED and anxiety. Together with the multi-tissue expression validation, these findings provide a hypothesis-generating framework for further investigating the potential interplay between ocular surface inflammation and anxiety-related neuroimmune alterations, rather than establishing a bidirectional causal relationship.

Nonetheless, several limitations must be acknowledged. (1) The core mechanisms proposed for DED-anxiety comorbidity necessitate further empirical validation in dedicated *in vitro* models. Moreover, the prognostic significance and translational potential of the identified comorbid genes require further elucidation. (2) For the immune infiltration correlation analyses between hub genes and immune cell fractions, raw *P*-values were used without FDR correction for multiple comparisons; therefore, these findings should be interpreted as exploratory and hypothesis-generating, requiring validation in independent cohorts. (3) Several limitations of the animal model should be acknowledged. The BAC-induced model primarily results from direct epithelial toxicity and may not fully replicate immune-mediated forms of DED, nor fully capture the heterogeneity of human comorbid conditions. The absence of DED-only and CUMS-only control groups prevents us from determining whether the observed changes are specific to comorbidity or independently associated with either condition alone. Additionally, the use of only female mice limits generalizability to males, as sex differences may affect ocular surface inflammation, stress susceptibility, and anxiety-like behaviors. Other potential confounding variables, such as circadian timing of behavioral testing and stress-related changes in locomotor activity or body weight, were not controlled. (4) RT-qPCR and Western blot analyses confirmed expression changes but did not establish functional causality.

Future studies using gene knockdown, overexpression, or pharmacological intervention are required. It also should include both male and female animals, perform sex-stratified analyses, and evaluate whether the identified hub genes and immune signatures exhibit sex-dependent expression patterns. The identified genes were derived from bioinformatic analyses and mouse experiments; therefore, validation in human clinical samples is necessary before considering their diagnostic or therapeutic potential. And research should prioritize the comprehensive functional characterization of hub genes and their involvement in critical signaling pathways, immune regulatory networks, and neuroimmune circuits pertinent to both DED and anxiety. The validation of the prognostic value of these candidate genes will necessitate large-scale, well-characterized clinical cohorts, which are essential for bridging the gap between bioinformatic predictions and clinical application.

## 5 Conclusion

In summary, this study identifies *BTBD1*, *BANK1*, *UBA3*, and *NEDD1* as potential genes linked to immune-inflammatory signatures underlying the comorbidity of DED and anxiety, utilizing integrated bioinformatic analyses and *in vivo* experimental validation. Experimental validation confirmed upregulation of these hub genes in corneal tissue, with *BANK1* uniquely elevated across all examined tissues. Although these genes emerged as consistently dysregulated candidates within the analyzed networks, the current findings do not establish causal or functional roles for these genes in the comorbid condition. Instead, our results offer a hypothesis-generating framework that may guide future experimental studies aimed at elucidating the mechanistic significance of shared immune pathways and neuroimmune interactions in the comorbidity of DED and anxiety.

## Data Availability

The original contributions presented in the study are included in the article/supplementary material. Further inquiries can be directed to the corresponding authors.
